# Perovskite Light-Emitting Diodes: Engineering, Stability, and Applications

**DOI:** 10.3390/mi17091102

**Published:** 2026-09-21

**Authors:** Zhengran He, Luke Schneider, Jiawei Gong, Jie Zhao, Kyeiwaa Asare-Yeboah

**Affiliations:** 1 Electrical and Computer Engineering, Penn State University Behrend, Erie, PA 16563, USA; 2 Mechanical Engineering, Penn State University Behrend, Erie, PA 16563, USA; 3 Computer Science and Software Engineering, Penn State University Behrend, Erie, PA 16563, USA

**Keywords:** perovskite light-emitting diodes, PeLEDs, dimensionality engineering, defect passivation, interface engineering, operational stability

## Abstract

Metal-halide perovskite light-emitting diodes (PeLEDs) have rapidly achieved external quantum efficiencies comparable to established organic and quantum-dot LEDs. Their narrow emission spectra, tunable bandgaps, high photoluminescence efficiencies, and low-temperature processing make them promising for displays, lighting, optical communication, and flexible electronics, although their commercialization remains limited by short operational lifetime, efficiency roll-off, unstable blue emission, ion migration, interfacial degradation, and poor large-area uniformity. This review provides a device-engineering-centered analysis connecting perovskite materials and processing conditions with charge injection, radiative recombination, optical extraction, and stability. It introduces the essential characteristics of 3D, 2D/quasi-2D, and nanocrystal perovskites and evaluates major engineering approaches, including composition and dimensionality control, crystallization regulation, defect passivation, transport-layer and interface modification, charge balancing, and optical outcoupling. An emphasis of this review is its comparison of the problems addressed by these approaches and their trade-offs among efficiency, spectral stability, lifetime, and manufacturing compatibility. Degradation under electrical operation is examined with attention to Joule heating, ion migration, charge accumulation, and interfacial reactions. Emerging patterned, reconfigurable, flexible, transparent, and communication devices are also discussed. Finally, the review identifies operational stability, efficient blue emission, scalable fabrication, high-resolution patterning, lead toxicity, and competition with OLEDs as the principal challenges for PeLED commercialization.

## 1. Introduction

### 1.1. Importance of Perovskite LEDs

Metal-halide perovskite light-emitting diodes (PeLEDs) have emerged as promising candidates for next-generation displays and lighting because they combine excellent light-emitting properties with relatively simple material processing [1,2]. Perovskites exhibit high photoluminescence efficiency, narrow emission spectra, and readily tunable bandgaps [3]. Their emission color can be adjusted across the visible and near-infrared regions by modifying their composition, dimensionality, or crystal size [4]. These characteristics enable PeLEDs to produce highly saturated colors, which are particularly valuable for high-resolution and wide-color-gamut displays.

Compared with conventional inorganic LEDs, perovskite emitters can generally be deposited through low-temperature solution-based processes, potentially reducing fabrication complexity and enabling large-area, flexible, and lightweight devices [5]. Compared with OLEDs, PeLEDs often exhibit narrower emission spectra and compositionally tunable emission, but these attributes do not by themselves establish overall technological superiority [6,7]. Their compatibility with printing and patterning techniques further supports their potential use in displays, optical communication, wearable electronics, and reconfigurable photonic systems. Although operational stability and manufacturing reproducibility remain major barriers [8,9,10], the rapid improvement in PeLED performance demonstrates the strong potential of perovskites as an important light-emitting semiconductor platform.

### 1.2. Advantages Compared with OLEDs and Quantum-Dot LEDs

Compared with organic light-emitting diodes (OLEDs), PeLEDs can exhibit narrower electroluminescence spectra, which can support high color purity and wide color gamuts [11,12]. Their emission wavelength can be adjusted by changing the halide composition, metal cation, organic or inorganic cation, or structural dimensionality. In contrast, tuning the emission of organic emitters often requires the design and synthesis of new molecules [13,14,15,16,17]. Perovskites also possess relatively high charge-carrier mobility and can transport both electrons and holes, which may facilitate charge injection and radiative recombination within the emitting layer.

PeLEDs share several advantages with quantum-dot LEDs (QLEDs) [18,19,20,21], including solution processability, tunable emission, and high photoluminescence efficiency. At laboratory scale, perovskite emitters can often be prepared through comparatively simple synthesis and deposition processes, but this does not establish lower manufacturing cost at commercial scale. Their defect-tolerant electronic structure [22,23] allows strong luminescence even when the material contains a considerable density of structural defects, potentially reducing the need for complex core–shell structures commonly employed in conventional quantum dots. Perovskites can also be fabricated as thin films, nanocrystals, or low-dimensional structures, providing considerable flexibility in controlling charge confinement, emission wavelength, and device architecture. These properties make PeLEDs an attractive platform for developing efficient and color-pure light-emitting devices, while commercial-scale cost, lifetime, and manufacturing maturity remain to be established relative to OLED and QLED technologies. In a technology-level comparison, PeLEDs are now competitive in peak efficiency and can offer narrow emission spectra, while OLEDs retain a substantially more mature manufacturing base and OLED/QLED platforms have longer-established operational-stability datasets; commercial-scale cost comparisons remain process- and scale-dependent.

### 1.3. Current Performance and Practical Limitations

The performance of perovskite light-emitting diodes has improved remarkably since the first room-temperature devices were reported. State-of-the-art PeLEDs now achieve external quantum efficiencies comparable to those of mature OLED and quantum-dot LED technologies. Peak EQEs above 25% have been demonstrated for red and green emission, while blue PeLEDs have exceeded 20% under optimized conditions. For example, reported benchmark EQEs have reached over 26% for red [24], 30% for green [25], and 21% for blue devices [26]. More recently, a peak EQE of 32.0% was achieved by accelerating radiative recombination in a formamidinium lead iodide emitting layer, with the EQE remaining above 30% at a current density of 100 mA cm^−2^ [27]. These results demonstrate that the fundamental efficiency of PeLEDs is no longer their only major limitation [28]. Hybrid perovskite-organic light-emitting architectures have reported external quantum efficiencies above 40%; however, because these devices rely on sensitized or hybrid emission rather than emission solely from the perovskite layer, they fall outside the primary scope of the PeLED benchmarks considered here [29]. Table 1 places representative visible red, green, and blue benchmarks in a common reporting framework; the 32.0% FAPbI_3_ result is listed separately as a near-infrared benchmark rather than as a visible red-display benchmark.

Considerable progress has also been made in brightness [30,31,32], operating voltage [33,34], and efficiency roll-off [35,36]. Effective defect passivation, improved charge balance, and suppression of Auger recombination have allowed some PeLEDs to maintain EQEs above 20% at high current densities [37,38]. This is particularly important because a high peak EQE measured at low brightness does not necessarily represent useful device performance. Display and lighting applications require devices to maintain their efficiency at practical luminance levels without excessive voltage, heat generation, or spectral change [39,40,41]. Active-matrix red, green, and blue PeLEDs with microsecond response times and EQEs of 22.7%, 26.2%, and 18.1%, respectively, have been demonstrated, showing the possibility of integrating PeLEDs into actively driven display systems [42]. Nevertheless, most record efficiencies are still obtained from small laboratory devices under carefully controlled conditions.

The performance of PeLEDs also varies considerably with emission color. Green and red devices are currently the most developed because efficient emission can be obtained from relatively stable bromide- and iodide-based compositions [43,44,45]. Blue PeLEDs remain more difficult because blue emission commonly requires either chloride-rich mixed-halide compositions or strong quantum confinement [46,47,48,49]. Mixed-halide materials can undergo electric-field-induced ion migration and phase segregation, causing the emission peak to shift during operation [50,51,52,53]. Strongly confined quasi-2D perovskites may contain multiple phases with different energy levels, leading to uneven charge transport and incomplete energy transfer [54,55]. Although a blue PeLED emitting at 487 nm has achieved an EQE of 23.3%, efficient deep-blue emission with appropriate color coordinates and long operational stability remains substantially more challenging [56]. Therefore, reporting a high EQE alone is insufficient, and emission wavelength, spectral stability, brightness, and efficiency roll-off must be considered together.

Operational stability is the most significant barrier to practical PeLED applications [57,58,59]. Perovskite materials are sensitive to moisture, oxygen, heat, and electrical bias [60]. During operation, injected charges and Joule heating can accelerate defect formation, ion migration, interfacial reactions, and structural decomposition. Mobile ions may accumulate near the transport-layer interfaces, modifying local electric fields and disrupting the balance between electron and hole injection. In addition, organic transport layers and metal electrodes can react with the perovskite or allow ions to diffuse across the device. These processes cause luminance decay, voltage drift, spectral changes, and eventual device failure. Some advanced PeLEDs have demonstrated greatly extended lifetimes, including a directly measured T_50_ of 18.67 h at a high initial luminance of 12,000 cd m^−2^ [61]. However, lifetime values are strongly dependent on initial luminance, current density, encapsulation, testing atmosphere, and the definition of device failure. Consequently, lifetime results from different studies cannot always be directly compared. No single application-independent lifetime threshold defines PeLED commercialization; meaningful comparison requires a stated failure criterion, initial luminance or current density, driving protocol, atmosphere or encapsulation condition, and an explicit distinction between directly measured and extrapolated lifetime [62].

Large-area fabrication presents another major challenge [63,64]. Record devices are typically produced by spin coating and have active areas of only a few square millimeters. Increasing the device area makes it more difficult to control film thickness, crystallization, surface roughness, and defect distribution [65]. Pinholes and local variations in film morphology can create current leakage and nonuniform emission. Various methods such as blade coating [66,67,68], printing [69,70], and thermal evaporation [71,72,73] have shown encouraging progress, but their performance generally remains below that of optimized small-area devices. Doctor-bladed PeLEDs have achieved an EQE of 16.1% [74], while blade-coated white and red devices have reached EQEs of 10.6% and 15.3%, respectively [75]. Uniform emission has also been demonstrated over areas up to 28 cm^2^, indicating progress toward large-area lighting applications. However, further improvements in coating uniformity, process tolerance, and device-to-device reproducibility are required. Scalable-processing studies in perovskite photovoltaics provide useful insight into coating uniformity and film formation, but electrically driven PeLEDs impose additional requirements for charge injection, emission uniformity, interfacial compatibility, and operational stability. PeLED-specific demonstrations therefore provide the more appropriate benchmark for device-scale performance.

High-resolution patterning and integration with transistor backplanes are also necessary for display applications [76,77,78]. Perovskite layers are easily damaged by the solvents, heat, radiation, and chemicals used in conventional photolithography. Patterning can introduce surface defects, remove passivating ligands, or alter the perovskite composition, thereby reducing luminescence efficiency. Although printing, transfer patterning, fine-mask evaporation, and localized treatment have been investigated, simultaneously achieving small pixel dimensions, high pixel density, uniform emission, and long lifetime remains difficult. Reconfigurable devices, such as writable and wipeable PeLEDs, provide an alternative approach by modifying emission locally after device fabrication. However, their pattern resolution, rewriting endurance, processing speed, and compatibility with active-matrix addressing must still be established. Patterning approaches developed for perovskite detectors and imaging devices provide useful processing concepts, but emitter applications additionally require preservation of electroluminescence efficiency, color stability, and charge injection after patterning.

Finally, most high-performance PeLEDs contain lead, raising concerns about material toxicity, leakage, disposal, and environmental impact [79,80]. Lead-free perovskites based on tin, bismuth, antimony, or other metals have been investigated, but they generally exhibit lower efficiency or poorer stability. Tin-based perovskites, for example, are vulnerable to oxidation from Sn^2+^ to Sn^4+^, which increases defect density and nonradiative recombination [81,82,83]. Effective encapsulation, reduced material usage, lead-capture layers, and recycling strategies may lower the environmental risk, but these approaches add complexity to manufacturing. Therefore, the future of PeLED technology will depend not only on achieving higher peak efficiency, but also on combining efficiency with operational stability, scalable patterning, reproducibility, and responsible material management.

### 1.4. Scope of the Review

This review focuses on the material and device engineering of perovskite light-emitting diodes, with particular attention to the factors that determine electroluminescence efficiency, operational stability, and practical applicability. The discussion is limited to the fundamental concepts required to understand device behavior rather than providing an exhaustive account of perovskite chemistry. Accordingly, 3D, 2D/quasi-2D, and nanocrystal perovskites are introduced in terms of their carrier confinement, phase distribution, charge transport, emission control, and defect-mediated recombination. The main emphasis of this review is placed on engineering strategies that directly affect PeLED performance, including composition and dimensionality control, film morphology and crystallization regulation, defect passivation and additive engineering, transport-layer and interface modification, charge-injection balance, and optical outcoupling. Representative studies are compared according to the problems they address and their effects on EQE, efficiency roll-off, emission stability, operating voltage, and device reproducibility. The trade-offs associated with each approach, including restricted charge transport, complex processing, and limited compatibility with large-area fabrication, are also considered. Device degradation is discussed specifically under electrical operation, including Joule heating, ion migration, charge accumulation, interfacial reactions, and electrode degradation. General environmental stability of isolated perovskite films is considered only when it directly influences an operating device. Emerging patterned and reconfigurable displays, flexible and transparent PeLEDs, and optical-communication devices are subsequently examined to illustrate the expanding functionality of this technology. Finally, the review identifies operational lifetime, efficient and stable blue emission, large-area uniformity, high-resolution patterning, lead toxicity, and competition with OLEDs as the principal challenges facing practical PeLED development. Accordingly, this review synthesizes material and device metrics reported in the cited primary literature rather than presenting newly synthesized materials or independently measured properties.

## 2. Materials and Emission Mechanisms

The performance of perovskite light-emitting diodes is governed by the interaction among material dimensionality, charge injection, radiative recombination, bandgap control, and defect formation. This section introduces only the concepts needed to understand how these factors affect device efficiency, emission color, and stability.

### 2.1. 3D, 2D/Quasi-2D, and Nanocrystal Perovskites

Three-dimensional perovskites generally have the ABX_3_ structure, where A is a monovalent cation, B is commonly Pb^2+^ or Sn^2+^, and X is Cl^−^, Br^−^, or I^−^. Their continuous network of corner-sharing BX_6_ octahedra supports efficient electron and hole transport [84,85]. However, relatively weak carrier confinement allows charges to migrate toward grain boundaries and interfaces, where nonradiative losses may occur. Therefore, high-performance 3D PeLEDs usually require small grains, uniform film coverage, and effective defect passivation. With appropriate control of crystallization and recombination, 3D perovskites can support very high efficiencies, including a reported peak EQE of 32.0% for FAPbI_3_-based devices [27]. On the other hand, two-dimensional perovskites contain bulky organic cations that separate the inorganic metal-halide framework into quantum wells [86,87,88]. The number of inorganic layers is represented by *n*, with *n* = 1 corresponding to a strictly 2D structure. These materials exhibit strong quantum and dielectric confinement, which increases exciton binding energy and favors radiative recombination. However, the insulating organic spacer layers can impede vertical charge transport.

Quasi-2D perovskites contain domains with different *n* values and offer a balance between the strong confinement of 2D structures and the better transport of 3D perovskites [89,90,91,92]. Energy can transfer from low-*n*, wide-bandgap domains to high-*n*, narrow-bandgap domains, concentrating carriers in the emitting regions. This energy-funneling process can enhance light emission, but an uncontrolled phase distribution may create transport barriers, trap carriers, and broaden the emission spectrum. Controlling the thickness, orientation, and distribution of the quantum wells is therefore essential. Reduced-dimensional PeLEDs with a controlled phase distribution have achieved EQEs above 25% [93].

Perovskite nanocrystals, including quantum dots, nanocubes, and nanoplatelets, provide additional carrier confinement and precise emission control [94,95,96,97]. Their high surface-to-volume ratio allows effective surface passivation and high photoluminescence quantum yield. However, organic ligands used to stabilize the nanocrystals are often electrically insulating. Long ligands improve colloidal stability but restrict charge transport between neighboring particles, whereas ligand removal may expose surface defects and destabilize the nanocrystals. Nanocrystal PeLED engineering therefore requires a careful balance among surface passivation, film stability, and electrical conductivity.

**Figure 1:**
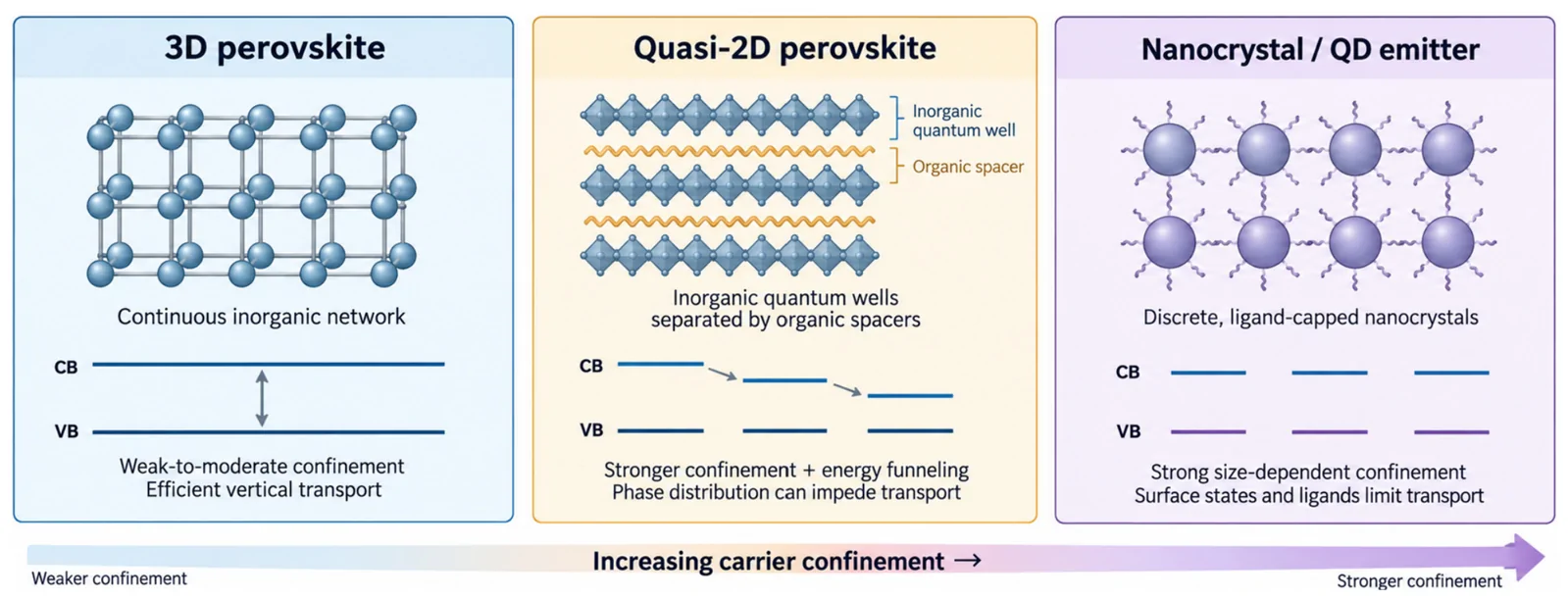
Conceptual comparison of 3D, quasi-2D, and nanocrystal PeLED emitters. Continuous 3D networks favor carrier transport but provide weaker confinement; quasi-2D structures confine carriers within inorganic quantum wells separated by organic spacers and rely on controlled band offsets for energy funneling; nanocrystals provide strong size-dependent confinement but introduce surface states and ligand-mediated transport barriers. Band diagrams are schematic and not to scale. The graphical elements in this conceptual schematic were generated using generative AI (ChatGPT 5.6–Sol) and subsequently reviewed and revised by the authors.

Figure 1 summarizes the contrasting confinement and transport landscapes of the three emitter classes. Three-dimensional perovskites provide continuous transport pathways with comparatively weak confinement; quasi-2D materials introduce quantum-well band offsets that favor energy funneling but can impede vertical transport; and nanocrystals provide strong size-dependent confinement while increasing the importance of surface states and ligand-mediated transport barriers.

### 2.2. Charge Injection and Radiative Recombination

A typical PeLED contains a perovskite emitting layer between electron- and hole-transport layers. Under an applied voltage, electrons are injected from the cathode through the electron-transport layer, while holes are injected from the anode through the hole-transport layer. These carriers enter the perovskite and recombine to generate photons. Efficient operation requires balanced electron and hole injection. If one carrier is injected much more efficiently than the other, excess charges accumulate in the emitting layer or at its interfaces. This accumulation increases leakage current, interfacial quenching, ion migration, and nonradiative recombination. The energy levels, conductivity, mobility, and thickness of the transport layers must therefore be selected to minimize injection barriers and place the recombination zone inside the perovskite rather than near a quenching interface. The EQE of a PeLED is primarily determined by charge-injection balance, the fraction of electron–hole pairs that recombine radiatively, and the fraction of generated light that escapes from the device. At low current density, radiative efficiency may be limited by traps and unbalanced injection. At high current density, Auger recombination, Joule heating, and electric-field-driven ion migration can cause efficiency roll-off [98,99,100]. Consequently, a high peak EQE does not necessarily indicate good practical performance. Efficient devices must maintain radiative recombination over a broad range of current densities and luminance levels.

### 2.3. Bandgap and Emission-Color Control

The emission color of metal-halide perovskites can be adjusted through composition and structural confinement [101]. Halide composition provides the most direct control: chloride-rich perovskites generally produce blue emission, bromide-based materials produce green emission, and iodide-rich materials produce red or near-infrared emission. Mixing halides allows intermediate wavelengths to be obtained. Changes to the A- and B-site ions can also modify the crystal structure and electronic bandgap, although their influence on color is generally less direct than that of the halide composition. Dimensionality and crystal size provide additional control. Reducing the number of inorganic layers in quasi-2D perovskites strengthens quantum confinement and increases the bandgap, shifting emission toward shorter wavelengths. Similarly, reducing nanocrystal dimensions can produce a blue shift when their size approaches the exciton Bohr radius. These approaches are particularly useful for blue emission because they can reduce the amount of chloride required.

Color tuning frequently introduces a tradeoff between spectral control and stability [102]. Mixed-halide perovskites can undergo electric-field-induced phase segregation, in which bromide- and iodide- or chloride-rich regions form during operation. This changes the local bandgap and causes the emission wavelength to shift. Quasi-2D materials may also contain an uncontrolled distribution of phases with different bandgaps, while nanocrystals can undergo halide exchange or aggregation. Stable color emission therefore requires control of ion migration, phase distribution, surface chemistry, and interfacial electric fields. Stronger ligand binding has been shown to suppress halide segregation and improve the bandgap stability of mixed-halide nanocrystal LEDs [103].

### 2.4. Defects and Nonradiative Recombination

Although metal-halide perovskites are often described as defect tolerant, defects still strongly affect PeLED performance [104,105,106]. Halide vacancies, undercoordinated lead ions, grain boundaries, and imperfect surfaces can introduce electronic states that trap electrons or holes. Shallow defects may have a limited effect on emission, but deep traps provide pathways for carriers to lose energy without producing photons. Because quasi-2D and nanocrystal emitters contain large interfacial or surface areas, their performance is particularly sensitive to surface defects. Nonradiative recombination can occur within the perovskite bulk, at grain boundaries, at nanocrystal surfaces, or at interfaces with the transport layers. Interfacial losses are especially important when the recombination zone is located close to a transport layer containing quenching sites. Under continuous operation, ion migration and interfacial chemical reactions may create additional defects, causing the efficiency and luminance to decrease over time [107,108]. Passivation molecules and additives are commonly used to coordinate undercoordinated metal ions, fill halide vacancies, control crystallization, and protect grain boundaries [109,110,111]. However, excessive or poorly selected passivators can inhibit charge transport or disturb the energy-level alignment. Effective passivation must therefore reduce trap-assisted recombination without introducing electrically insulating barriers. This balance between defect suppression and charge transport is one of the central principles underlying material and interface engineering in PeLEDs.

## 3. Material and Device Engineering

### 3.1. Composition and Dimensionality Engineering

Composition and dimensionality engineering are among the most direct methods for controlling the emission wavelength, radiative efficiency, charge transport, and stability of PeLEDs. The composition determines the electronic structure and crystallization behavior of the perovskite, whereas dimensionality controls the extent of carrier confinement and the pathway through which charges reach the emitting regions. Effective engineering requires these two factors to be considered together because a composition that provides the desired bandgap may not necessarily produce a stable film or efficient device.

In the ABX_3_ perovskite structure, the X-site halide composition has the strongest influence on the bandgap [112,113,114]. Iodide-rich perovskites are normally used for red and near-infrared emission, bromide-based perovskites for green emission, and chloride incorporation for blue emission. Mixing halides enables continuous wavelength tuning, but it can also produce compositional inhomogeneity. Under electrical bias, mobile halide ions may redistribute and form regions with different bandgaps. Injected carriers then preferentially recombine in the lower-bandgap regions, causing the electroluminescence spectrum to shift. This field-induced phase segregation is particularly problematic for mixed Br/Cl blue emitters and mixed Br/I red emitters. These composition trends provide a useful basis for bandgap control, but spectral stability under electrical operation also depends on ion migration, interfacial fields, and device architecture; electroluminescent measurements under bias are therefore required to assess stability directly.

Halide engineering must therefore control not only the average composition but also its spatial uniformity and resistance to ion migration. Improving precursor mixing, strengthening ionic interactions, and reducing halide-vacancy density can suppress phase segregation [115,116]. For example, cationic surfactants have been used to improve halide homogeneity in precursor solutions, producing spectrally stable blue-emitting 3D perovskites with high brightness [117]. This result shows that spectral stability begins with compositional uniformity during film formation rather than only with the final device structure. The A-site cation does not contribute strongly to the electronic states at the band edges, but it affects lattice distortion, phase stability, crystallization, and defect formation. Methylammonium, formamidinium, and cesium are commonly used individually or in combination. Formamidinium-based perovskites can provide relatively narrow bandgaps and favorable radiative properties, while cesium can improve thermal stability and modify crystallization. Mixed A-site systems often combine these advantages, but introducing multiple cations can also make crystallization less uniform. The cation ratio must therefore be optimized to obtain the desired crystal phase without producing excessive compositional disorder. Trends reported in photovoltaic, photocatalytic, or perovskite-inspired systems can help identify possible composition-structure relationships, but their effects on electroluminescent performance must be evaluated under PeLED operating conditions.

**Figure 2:**
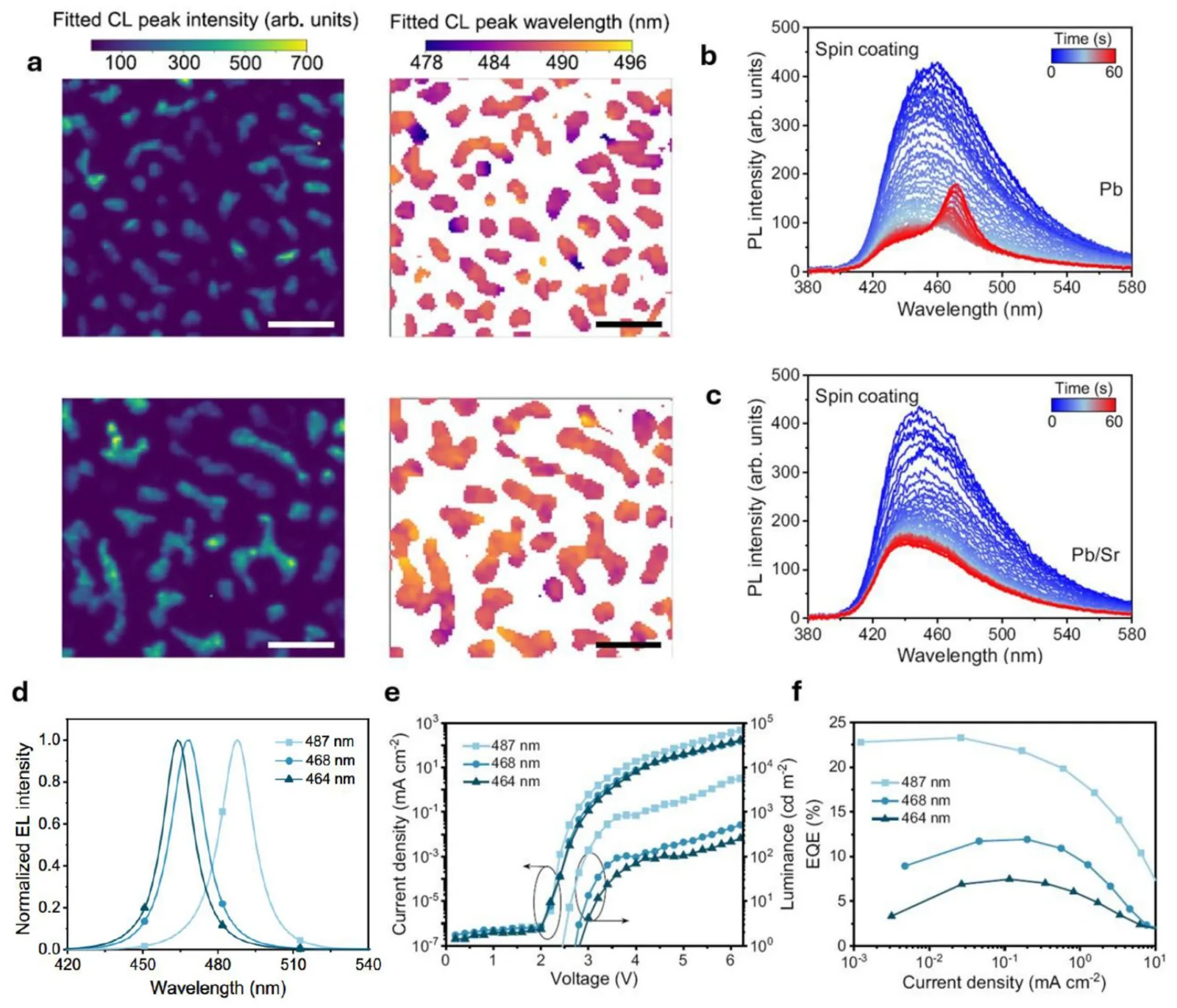
All-site alloying for crystallization control and efficient blue three-dimensional perovskite LEDs. (**a**) Fitted cathodoluminescence (CL) peak-intensity and peak-wavelength maps of Pb-only (top) and Pb/Sr-alloyed (bottom) perovskite films; scale bars, 700 nm. (**b**,**c**) In situ photoluminescence spectra recorded during spin-coating of the Pb-only and Pb/Sr-alloyed films, respectively. (**d**) Normalized electroluminescence spectra of devices emitting at 487, 468, and 464 nm. (**e**) Current density and luminance versus voltage and (**f**) external quantum efficiency versus current density for the three emission wavelengths. Reproduced from Reference [56] under CC BY-NC-ND 4.0, with permission from Springer Nature.

Chen et al. applied an all-site alloying strategy to control the disordered crystallization of multicomponent blue 3D perovskites [56]. Partial Pb/Sr substitution delayed rapid film formation and facilitated the sequential incorporation of FA and Cs cations. The CL maps show that the Pb/Sr-alloyed film had a more uniform emission wavelength than the Pb-only film, with the standard deviation decreasing from 1.9 to 1.5 nm (Figure 2a). During spin-coating, the Pb-only film developed a distinct perovskite emission peak near 469 nm after approximately 35 s, whereas no corresponding perovskite emission appeared in the Pb/Sr film, which the authors interpreted as evidence that Sr retarded the initial crystallization process (Figure 2b,c). This slower, stepwise growth was accompanied by a reduction in the trap density from 2.2 × 10^15^ to 4.2 × 10^14^ cm^−3^. The resulting device emitted at 487 nm with a peak EQE of 23.3% and a maximum luminance of approximately 5700 cd m^−2^. Subsequent halide exchange shifted the emission to 468 and 464 nm, with peak EQEs of 11.9% and 7.5%, respectively (Figure 2d–f). Together, these measurements support the authors’ interpretation that coordinated A-, B-, and X-site engineering can regulate crystallization, suppress defects, and tune blue emission.

The B-site metal determines much of the band structure and carrier transport [118,119,120]. Lead-based perovskites currently provide the best combination of defect tolerance, charge mobility, and emission efficiency. Partial substitution with metals such as Sr^2+^, Zn^2+^, Mn^2+^, or other compatible ions may modify crystallization, reduce trap formation, or stabilize the lattice. However, excessive substitution can create secondary phases, increase energetic disorder, or interrupt the metal-halide framework. B-site alloying should therefore be treated as a controlled modification rather than a simple replacement of lead. Fully lead-free emitters based on Sn^2+^, Bi^3+^, or Sb^3+^ remain attractive for environmental reasons, but their device efficiencies generally lag behind lead-based PeLEDs. In particular, Sn^2+^ readily oxidizes to Sn^4+^, producing high defect densities and strong nonradiative recombination. Because these effects depend strongly on composition and device architecture, B-site substitutions must be evaluated under electroluminescent operation, where changes in charge transport, spectral stability, and efficiency may differ from those observed in other perovskite systems.

**Figure 3:**
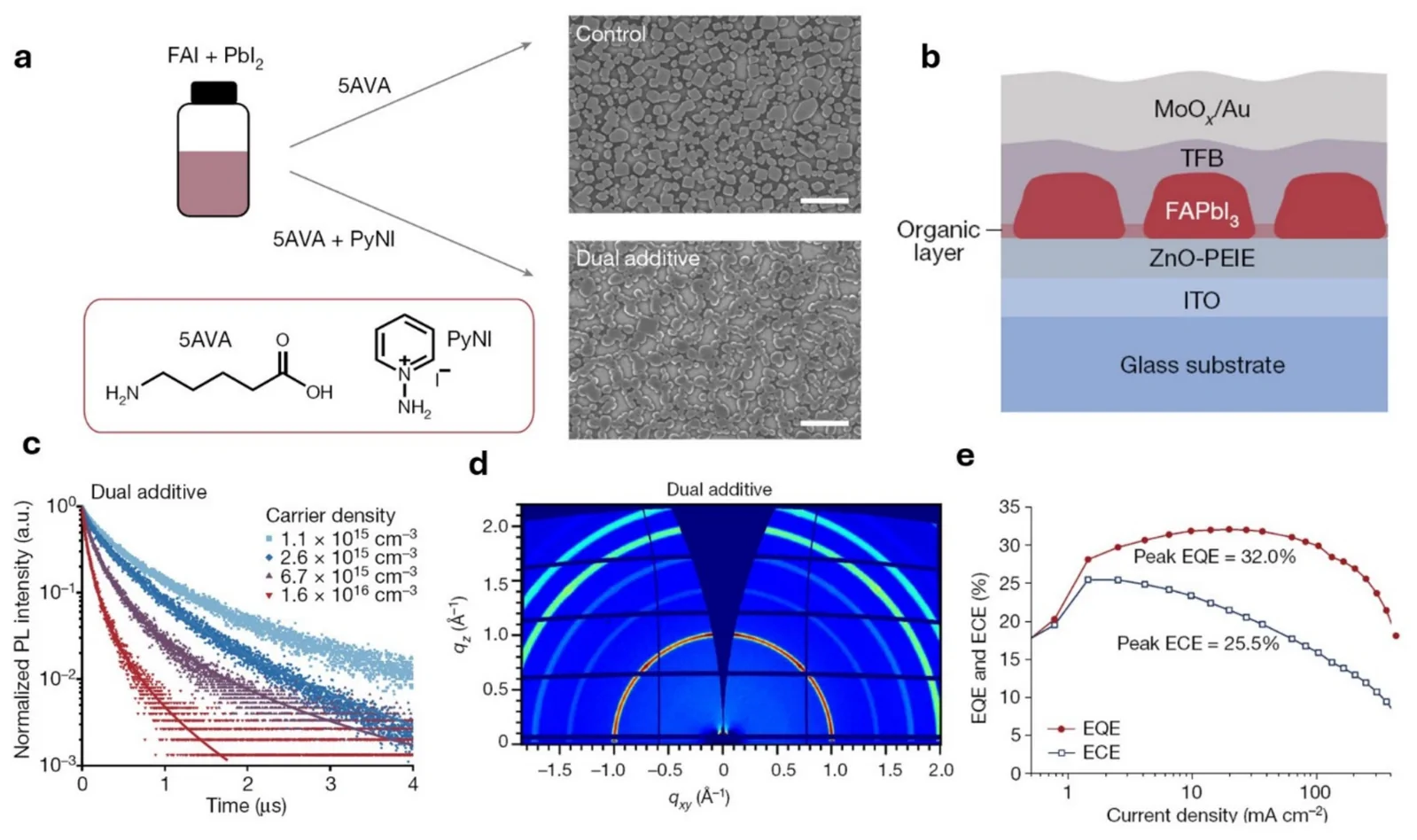
Dual-additive crystallization and radiative-recombination engineering of three-dimensional FAPbI_3_ PeLEDs. (**a**) Preparation of the control film using 5-aminovaleric acid (5AVA) and the dual-additive film using 5AVA and 1-aminopyridinium iodide (PyNI), together with their molecular structures and surface SEM images. Scale bars: 1 μm. (**b**) Device architecture consisting of glass/ITO/ZnO–PEIE/FAPbI_3_/TFB/MoO_x_/Au. (**c**) Carrier-density-dependent time-resolved photoluminescence decays of the dual-additive film. (**d**) Two-dimensional GIWAXS pattern of the dual-additive film measured at an incidence angle of 0.20°. (**e**) External quantum efficiency and energy-conversion efficiency as functions of current density, showing peak values of 32.0% and 25.5%, respectively. Reproduced from Reference [27] under CC BY 4.0, with permission from Springer Nature.

Three-dimensional perovskites provide efficient charge transport but generally exhibit weaker carrier confinement than low-dimensional emitters. Li et al. addressed this limitation using a dual-additive crystallization strategy for FAPbI_3_ [27]. As shown in Figure 3a, the control formulation contained 5-aminovaleric acid (5AVA), whereas the dual-additive formulation contained both 5AVA and 1-aminopyridinium iodide (PyNI). Although the two films exhibited similar discrete submicrometre morphologies, the additives modified the crystallization pathway and internal phase structure. The carrier-density-dependent photoluminescence decays in Figure 3c are consistent with density-dependent radiative recombination, while comparison with the control and kinetic analysis in the original study attributed the faster decay to enhanced excitonic and bimolecular radiative processes. This interpretation was supported by an increase in photoluminescence quantum efficiency from approximately 70% to 96%. GIWAXS analysis, represented by Figure 3d and supported by the depth-dependent measurements in the original study, showed a higher proportion of tetragonal FAPbI_3_ in the dual-additive film. The corresponding exciton binding energy increased from approximately 3.9 to 13.9 meV; the original study linked this increase to enhanced radiative recombination. Using the device architecture shown in Figure 3b, the near-infrared PeLED achieved a peak EQE of 32.0% and a peak energy-conversion efficiency of 25.5%, while retaining an EQE of approximately 30% at 100 mA cm^−2^ (Figure 3e). These results support the interpretation that engineering the crystallization pathway and crystal phase can accelerate radiative recombination and suppress efficiency roll-off without relying solely on defect passivation.

Two-dimensional perovskites provide stronger exciton confinement but often suffer from poor vertical charge transport because of their insulating organic spacer layers [121,122]. For this reason, strictly 2D perovskites are less commonly used alone in high-performance PeLEDs. Quasi-2D perovskites are generally preferred because they contain a mixture of quantum wells with different thicknesses. Low-*n* phases provide strong confinement, while higher-*n* phases support better charge transport and usually act as the final emitting regions. The performance of quasi-2D PeLEDs depends more strongly on the actual phase distribution than on the nominal *n* value calculated from the precursor ratio. Randomly distributed phases can create discontinuous energy-transfer pathways, trap carriers, and broaden the emission spectrum. An excessive amount of low-*n* material may impede charge injection, whereas an excessive amount of high-*n* material weakens confinement. The engineering objective is therefore not necessarily to eliminate the multiphase structure, but to create a controlled energy landscape that directs carriers toward a limited population of efficient emitting domains. Ma et al. investigated this approach using tris(4-fluorophenyl)phosphine oxide (TFPPO), a bifunctional additive introduced through the antisolvent [93]. In control films, rapid PEA diffusion produces polydisperse QWs and vacancy defects, whereas TFPPO forms hydrogen bonds with PEA to slow its diffusion and uses its P=O group to passivate undercoordinated Pb sites. The transient-absorption maps show several bleach features in the control film, corresponding to QWs with different thicknesses, while the TFPPO-treated film exhibits a narrower response dominated by *n* = 5+ QWs near 495 nm. The authors associated this more uniform energy landscape with reduced energetic disorder and more efficient carrier funneling toward emitting regions. Using the device structure and energy-level alignment, the resulting PeLEDs achieved a peak EQE of 25.6% and an average EQE of 22.1 ± 1.2% across 40 devices. They also exhibited a directly measured operational half-life of 115 min at an initial luminance of 7200 cd m^−2^ under a constant current density of 8 mA cm^−2^, consistent with the interpretation that controlling QW distribution and defect density can improve both efficiency and stability.

Spacer design can also improve spectral and structural stability. Increasing ligand conjugation and cross-sectional area has been shown to inhibit ion diffusion and suppress phase disproportionation in quasi-2D perovskites. This produced deep-red PeLEDs with a peak EQE of 26.3% and improved wavelength stability [123]. For blue quasi-2D devices, controlling the phase distribution is especially important because random low-*n* and high-*n* domains can cause spectral shifts and nonradiative losses. Modification of the underlying injection layer with CsCl has been shown to rearrange quasi-2D phase formation while improving charge injection, resulting in sky-blue emission at 486 nm and a peak EQE of 16.07% [124]. This observation suggests that dimensionality is influenced by the substrate and interface, not only by the perovskite precursor composition.

In nanocrystal PeLEDs, dimensionality is controlled through particle size and shape [125,126]. Quantum dots and thin nanoplatelets provide strong confinement and allow emission tuning without requiring large changes in halide composition, which can be advantageous for blue emission because it may reduce the phase-segregation problem associated with chloride-rich mixed-halide materials [127,128]. However, stronger confinement increases the surface-to-volume ratio and makes emission more sensitive to surface defects and ligand loss. Very small nanocrystals may also exhibit inefficient charge transport and strong Auger recombination at high carrier density. Size control must therefore be combined with ligand and surface engineering.

Overall, no single dimensionality is optimal for every PeLED. Three-dimensional perovskites generally provide the most direct charge transport and are particularly effective for green and near-infrared emission, whereas quasi-2D and nanocrystal emitters provide stronger carrier confinement and greater control of blue emission. These benefits are accompanied by less uniform phase distributions, insulating organic components, or ligand-limited transport. Consequently, the most meaningful performance improvements are obtained when dimensionality is selected for the required emission color and then optimized together with defect density, charge transport, spectral stability, and film uniformity. From a manufacturing perspective, 3D compositions are generally easier to process over large areas, while quasi-2D and nanocrystal systems require tighter control of phase or particle distribution. Efficiency records are high across all three emitter classes, but spectral stability and operational lifetime depend strongly on phase control, surface chemistry, interfaces, and test conditions rather than on dimensionality alone; Table 2 summarizes these trade-offs.

### 3.2. Film Morphology and Crystallization Control

The morphology of the perovskite emitting layer directly influences current leakage, charge injection, radiative recombination, and device reproducibility [130,131]. An effective PeLED film should provide complete substrate coverage, limited pinhole formation, controlled grain dimensions, low interfacial roughness, and uniform thickness. These requirements are closely connected because all of them are determined by nucleation, crystal growth, solvent evaporation, and phase transformation during film formation. Pinholes are particularly harmful in PeLEDs because they can create direct electrical contact between the electron- and hole-transport layers [132,133]. These local shunting pathways increase leakage current, reduce the fraction of charges recombining in the perovskite, and may produce localized heating or premature breakdown. A rough perovskite surface can also prevent uniform deposition of the next transport layer, leading to variations in the electric field and current density [134,135]. Therefore, complete coverage and a suitable surface roughness are often more important than simply maximizing crystallinity.

**Figure 4:**
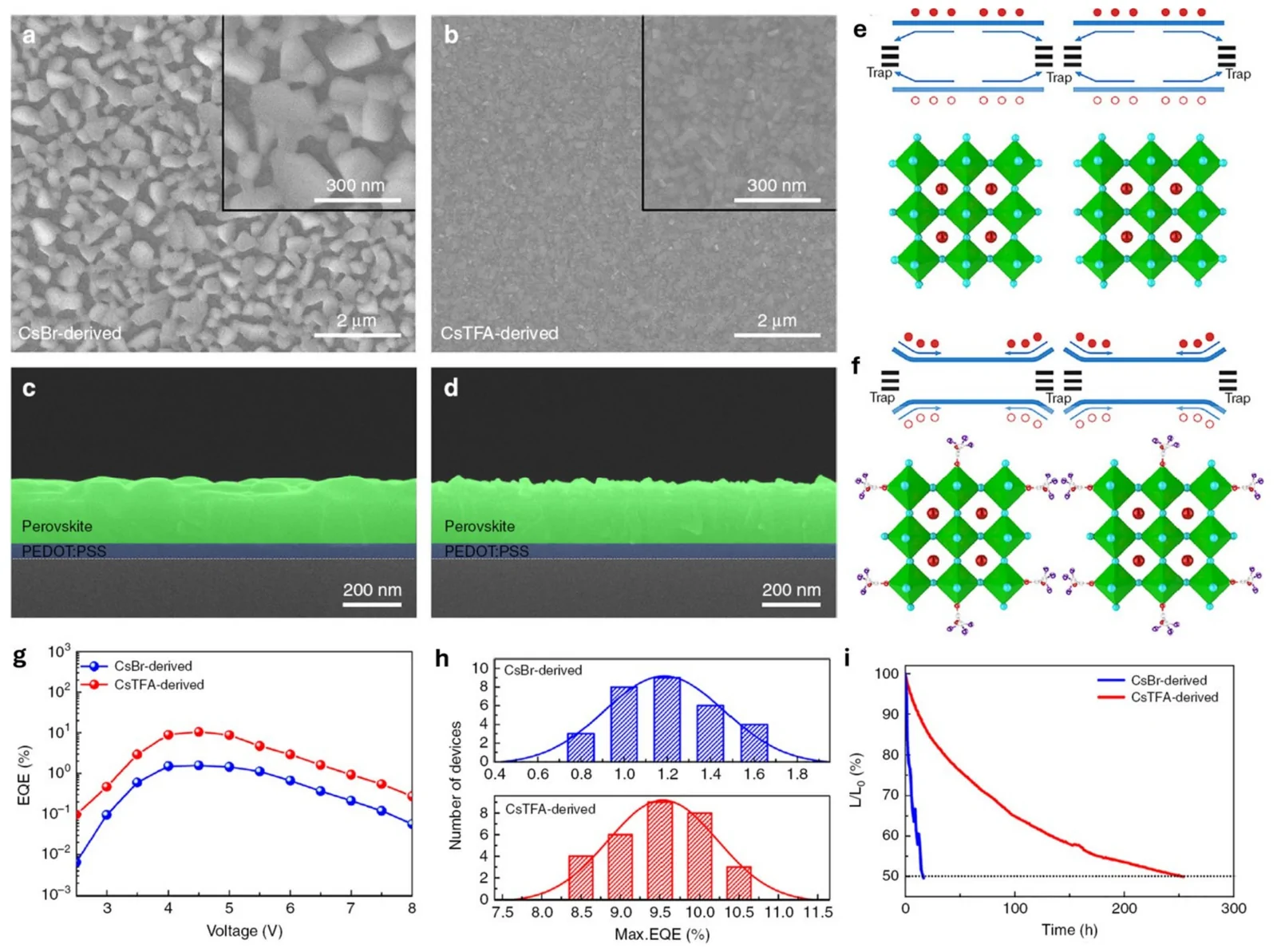
Trifluoroacetate-mediated morphology and grain-boundary engineering in CsPbBr_3_ PeLEDs. (**a**,**b**) Top-view field-emission scanning electron microscopy images of CsBr- and CsTFA-derived films, respectively; the insets show higher-magnification images. (**c**,**d**) Corresponding cross-sectional images of the films deposited on PEDOT:PSS. (**e**,**f**) Schematics of carrier trapping at unpassivated grain boundaries in the CsBr-derived film and carrier redistribution away from TFA-passivated boundaries in the CsTFA-derived film. (**g**) External quantum efficiency versus voltage, (**h**) distributions of maximum external quantum efficiency across 30 devices, and (**i**) normalized operational luminance of CsBr- and CsTFA-derived PeLEDs. The lifetime measurements were performed in air using epoxy-encapsulated devices at an initial luminance of 100 cd m^−2^. Reproduced from Reference [140] under CC BY 4.0, with permission from Springer Nature.

The optimal grain structure for PeLEDs differs from that commonly desired for perovskite solar cells. In solar cells, large grains are generally preferred because fewer grain boundaries reduce recombination during long-range carrier extraction [136,137,138]. In PeLEDs, as carriers should recombine radiatively within the emitting layer rather than travel over long distances, smaller grains can provide spatial confinement, increase the probability of electron–hole capture, and prevent carriers from reaching quenching interfaces [139]. However, reducing the grain size also increases the total grain-boundary area, which can introduce traps and ion-migration pathways. Small grains are therefore beneficial only when their surfaces and boundaries are effectively passivated. Wang et al. reported a case in which grain-size reduction was combined with effective grain-boundary passivation [140]. Replacing CsBr with cesium trifluoroacetate (CsTFA) altered the precursor chemistry and increased the nucleation density, reducing the average CsPbBr_3_ grain size from approximately 300 to 60 nm. Consequently, the poorly covered CsBr-derived film was transformed into a smooth and pinhole-free CsTFA-derived film while maintaining a thickness of approximately 200 nm (Figure 4a–d). The original study attributed the performance change to TFA^−^ coordination with surface Pb2^+^ sites and grain-boundary passivation, which was proposed to create wider-bandgap boundary regions that redirect carriers toward the grain interiors and suppress nonradiative recombination and ion migration (Figure 4e,f). These improvements increased the peak EQE from 1.6% to 10.5%, while the average maximum EQE across 30 devices increased from 1.2% to 9.4% (Figure 4g,h). At an initial luminance of 100 cd m^−2^, the directly measured operational half-life increased from 15 h for the CsBr-derived device to more than 250 h for the CsTFA-derived device (Figure 4i), consistent with a combined role for morphology control and boundary passivation.

Solution-processed perovskites crystallize rapidly because of their ionic nature [141,142]. As the wet precursor film dries, solvent evaporation increases the precursor concentration until supersaturation and nucleation occur. The number of nuclei and their subsequent growth determine the final grain size, coverage, and roughness. If nucleation is too slow or spatially nonuniform, isolated large crystals and uncovered regions can form. If nucleation is excessively rapid, the film may contain disordered crystallites, incomplete phase conversion, and a high density of boundaries. The objective is thus to generate sufficient and uniformly distributed nuclei while maintaining enough time for the formation of a continuous film. Precursor concentration, solvent coordination strength, coating speed, antisolvent timing, annealing temperature, and ambient conditions all influence this process. Strongly coordinating solvents can form intermediate complexes with the metal-halide precursor and delay crystallization, whereas rapidly evaporating solvents can produce abrupt supersaturation. Antisolvent treatment accelerates nucleation by reducing precursor solubility, but small changes in the dripping time or volume may produce substantial morphological differences. Thermal annealing then removes residual solvent and promotes phase conversion, although excessive temperature or annealing time can cause grain coarsening, phase redistribution, or material decomposition.

Additive engineering is widely used to make this crystallization process more controllable [143,144]. Lewis bases can coordinate with undercoordinated Pb^2+^ and modify the precursor-intermediate phases [145,146]. Organic ammonium salts can regulate nucleation, restrict crystal growth, and passivate the resulting grain surfaces [147,148]. Polymers can improve solution viscosity, substrate wetting, and film continuity, while surfactants can reduce surface tension and suppress dewetting [149,150]. As transferable background rather than direct PeLED evidence, solution-processed organic-semiconductor studies show that polymer additives and crystallization control can modify molecular orientation, grain-boundary structure, and carrier-transport pathways [151,152,153,154]. Although the material systems differ, these studies reinforce the importance of evaluating additives through both morphological and electrical measurements. A useful additive frequently performs several functions simultaneously, but its concentration must be carefully controlled. An insufficient amount may have little effect, while an excessive amount can leave an insulating residue that restricts charge transport. Substrate properties also affect nucleation and morphology. Surface energy and chemical functionality determine whether the precursor solution spreads uniformly or forms droplets and uncovered regions. The underlying transport layer can additionally interact with precursor ions and alter crystal orientation or phase distribution. For example, introducing a polyvinylpyrrolidone interlayer improved substrate wetting during CsPbBr_3_ deposition, while the addition of MABr further controlled crystallization and reduced pinhole formation [155]. This illustrates why the morphology of the perovskite layer cannot be optimized independently of the surface on which it is deposited. Film thickness must also be controlled. A film that is too thin may contain incomplete coverage and permit charge leakage. A film that is too thick increases operating voltage and may cause charges to accumulate before reaching the recombination zone. Thickness also influences the optical field and the fraction of generated light that escapes from the device. The preferred thickness therefore depends on carrier mobility, transport-layer energy alignment, and device architecture. Uniformity across the active area is more important than achieving a single nominal thickness at one measurement location.

**Figure 5:**
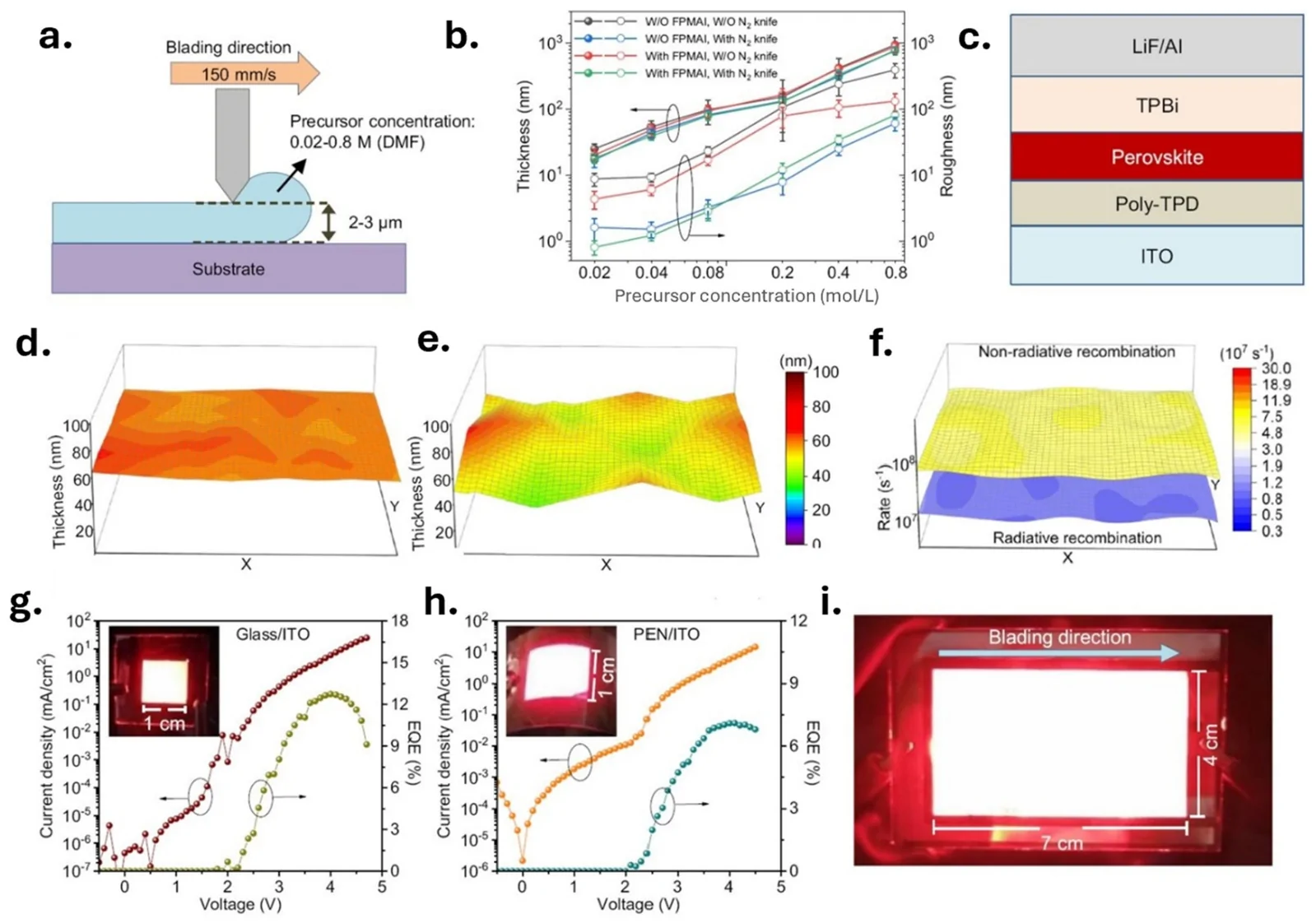
Low-temperature blade coating of uniform and large-area perovskite LEDs. (**a**) Schematic of blade coating at 150 mm s^−1^ using a 2–3 μm applicator gap. (**b**) Perovskite-film thickness and surface roughness as functions of precursor concentration, 4-fluorophenylmethylammonium iodide (FPMAI) addition, and N_2_-knife assistance. (**c**) Device architecture consisting of ITO/poly-TPD/perovskite/TPBi/LiF/Al. (**d**,**e**) Thickness distributions of 6 × 9 cm^2^ films prepared by blade coating and spin coating, respectively. (**f**) Spatially resolved radiative and nonradiative recombination rates of the blade-coated film. (**g**,**h**) Current-density and external-quantum-efficiency characteristics of 1 cm^2^ devices fabricated on rigid glass/ITO and flexible PEN/ITO substrates, respectively. (**i**) Photograph of a uniformly emitting 4 × 7 cm^2^ PeLED. Reproduced from Reference [74] under CC BY 4.0, with permission from Springer Nature.

Although smooth and compact films are generally desirable, intentionally structured morphology can sometimes improve light extraction. Amino-acid additives have been used to create spontaneously formed submicrometer structures while simultaneously passivating surface defects. These structures were reported to reduce optical trapping without strongly changing the emission spectrum or viewing-angle dependence, with a peak EQE of 20.7% [156]. This shows that the best morphology is not necessarily the smoothest possible surface. Morphology can be designed to improve both electrical recombination and optical outcoupling, provided that the structured film retains complete coverage and low defect density. Morphology control becomes more difficult as the device area increases. Spin coating can produce high-quality small-area films but depends on rapid radial solvent flow and discards much of the precursor. Blade coating, slot-die coating, and printing are more suitable for scalable fabrication, but their film formation is strongly affected by solution viscosity, coating speed, substrate temperature, and drying dynamics. Variations in these parameters can create thickness gradients, coffee-ring patterns, and nonuniform crystal growth. Chu et al. developed a low-temperature blade-coating process that controlled perovskite film formation through precursor dilution, FPMAI addition, and N_2_-knife-assisted drying [74]. Reducing the MAPbI_3_ precursor concentration increased the nucleation density and shortened the sol–gel transformation, while excess FPMAI suppressed grain growth and the N_2_ knife accelerated solvent evaporation (Figure 5a,b). With a 0.02 M precursor containing FPMAI, the film roughness decreased to approximately 0.8 nm. Thickness mapping across 6 × 9 cm^2^ films showed that blade coating produced substantially greater uniformity than spin coating, while recombination-rate mapping indicated spatially uniform radiative behavior and suppressed nonradiative loss (Figure 5d–f). Using the device structure shown in Figure 5c, the PeLEDs achieved a peak EQE of 16.1% over 0.04 cm^2^ and 12.7% over 1 cm^2^ on rigid glass. A 1 cm^2^ flexible device reached 7.1%, and uniform emission was demonstrated over 28 cm^2^ (Figure 5g–i). These results show that scalable PeLED fabrication requires coordinated control of coating dynamics, crystallization, and film thickness.

For nanocrystal PeLEDs, crystal synthesis and film formation can be separated, but the drying behavior of the nanocrystal ink remains important. Solvent evaporation, surface tension, and capillary or Marangoni flows can redistribute nanocrystals during coating, producing thickness variations and particle aggregation. A binary-solvent approach that controlled these flows produced uniform nanocrystal films over 54 cm^2^ and red devices with an EQE of 15.3%, together with uniform emission over an active area of 28 cm^2^ [75]. However, scalable nanocrystal deposition must also preserve surface ligands and avoid solvents that damage previously deposited layers. Vacuum deposition offers an alternative route with better thickness control and compatibility with established display manufacturing. Nevertheless, the arrival rate and mobility of vapor species can lead to rapid and uncontrolled nucleation, incomplete coverage, or broad grain-size distributions. Additives and co-evaporated passivators can regulate grain growth even in vacuum-processed films. For example, a fluorinated phosphine-oxide passivator reduced average grain size, eliminated pinholes, and improved the coverage of vacuum-deposited red-emitting perovskite films [157]. Thus, crystallization control remains necessary regardless of whether the film is formed from solution or vapor.

Morphological improvements should be supported by more than a single microscopy image. Scanning electron microscopy should evaluate coverage and grain distribution over multiple regions, while atomic force microscopy can quantify surface roughness. Cross-sectional imaging is needed to examine thickness and interface continuity. X-ray diffraction or grazing-incidence wide-angle X-ray scattering can identify crystal phases, orientation, and structural changes, whereas photoluminescence quantum yield and time-resolved measurements can determine whether the observed morphology actually reduces nonradiative recombination. Device statistics are also necessary because an improved champion device may result from random local variations rather than a reproducible crystallization process. Correlated changes in morphology, PL kinetics, and device EQE are mechanistically suggestive but are not independently conclusive; stronger causal assignment may require absolute PLQY, calibrated trap-density measurements, temperature-dependent spectroscopy, transient electroluminescence, or operando structural analysis.

Therefore, additive-controlled nucleation and crystallization provide the most direct improvements in film coverage, defect density, and device reproducibility, while intentionally structured morphologies can additionally enhance light extraction. The latter approach, however, produces rough or corrugated interfaces that may complicate transport-layer deposition and large-area uniformity. Blade coating offers greater manufacturing relevance than laboratory-scale spin coating, but its film thickness, drying dynamics, and device-to-device variation remain more difficult to control. The preferred strategy is therefore not simply to minimize grain size or roughness, but to establish a sufficiently wide processing window that preserves coverage, radiative efficiency, and uniformity across the intended device area.

### 3.3. Defect Passivation and Additive Engineering

Defect passivation is one of the most effective strategies for improving PeLED efficiency and stability [158,159,160]. Although metal-halide perovskites are often described as defect-tolerant semiconductors, their thin films still contain halide vacancies, undercoordinated metal ions, grain-boundary defects, and imperfect surface terminations. These defects can trap injected charges and promote nonradiative recombination. They can also facilitate ion migration and interfacial chemical reactions during device operation. The purpose of passivation is therefore not only to increase the initial photoluminescence efficiency but also to stabilize the perovskite under electrical and thermal stress. The most common passivation mechanism involves coordination between a Lewis-base functional group and an undercoordinated Pb^2+^ or Sn^2+^ ion [160,161]. Molecules containing phosphine oxide, sulfoxide, carbonyl, carboxyl, amino, or other electron-donating groups can provide electron density to these metal sites and reduce their ability to trap carriers. However, molecular structure matters as much as the presence of a functional group. Steric accessibility, molecular orientation, hydrogen bonding, and binding strength determine whether the passivating group can reach and remain attached to the defect. Rational control of these interactions has enabled near-infrared PeLEDs with an EQE of 21.6% and an EQE of 20.1% at a high current density of 200 mA cm^−2^ [37].

Halide vacancies can be passivated by supplying additional halide ions through metal-halide salts, ammonium halides, or other ionic additives, which can restore local coordination around the metal ions and reduce deep trap formation [162,163]. Halide-rich processing may also improve film stoichiometry and suppress the formation of metallic lead. However, excessive halide content can alter the bandgap, create secondary phases, or increase the concentration of mobile ions. The additive concentration must therefore be sufficient to compensate for vacancies without making the film compositionally or electrically unstable. Hydrogen bonding and ionic interactions provide another form of passivation [164,165,166]. Molecules containing ammonium, phosphonate, sulfonate, or related groups can interact with halide ions and organic cations, strengthening the local lattice and restricting molecular motion. This is especially important because the perovskite lattice is soft and dynamically disordered. Under electrical bias and elevated temperature, weakly bound ions can move or reorient, creating new defects even when the initial film is well passivated. Strong but reversible molecular interactions can suppress these fluctuations and reduce both nonradiative recombination and ion migration.

Surface passivation with bulky ammonium molecules can provide a physical barrier to ion movement [167,168]. The molecular chain length and binding configuration determine the effectiveness of this barrier. Phenylalkylammonium iodides with optimized chain lengths have been shown to reduce surface defects and suppress iodide migration in 3D perovskites. Devices treated with phenylpropylammonium iodide achieved an EQE of 17.5% and a directly measured T_50_ of 130 h under constant-current operation at 100 mA cm^−2^, as reported by Guo et al. [169]. The authors attributed the operational-stability improvement partly to restricted ionic motion rather than only to removal of static electronic traps. Passivating additives can be introduced directly into the precursor solution or applied after the perovskite film has formed. Precursor additives can reach the film interior and grain boundaries, allowing bulk and surface defects to be treated simultaneously. They can also coordinate with precursor species and modify nucleation, crystal growth, grain size, and phase formation. This multifunctionality can be beneficial, but it makes the mechanism more difficult to isolate. An apparent improvement in defect density may partly result from changes in morphology or dimensionality rather than direct chemical passivation.

Post-deposition treatments primarily target the upper surface and near-surface grain boundaries [170,171]. This allows the perovskite film to be formed before the passivator is introduced and may reduce unwanted effects on bulk crystallization. However, the treatment solvent must not dissolve or reconstruct the perovskite. An excessively thick passivation layer may also obstruct charge injection from the adjacent transport layer. Surface treatment therefore requires careful control of passivator concentration, solvent polarity, and treatment time. Defects can occur at both the upper and lower boundaries of the emitting layer. The buried interface between the perovskite and the underlying transport layer is formed during crystallization and cannot always be repaired through a top-surface treatment. Bilateral passivation, in which both interfaces are modified, can reduce carrier trapping and interfacial quenching throughout the complete emitting layer. In perovskite quantum-dot LEDs, phosphine-oxide molecules placed at both the upper and lower interfaces increased the film photoluminescence quantum yield from 43% to 79% and raised the device EQE from 7.7% to 18.7%. The reported T_50_ at 100 cd m^−2^ increased from approximately 0.8 to 15.8 h by extrapolation, whereas the directly measured T_50_ at an initial luminance of about 1000 cd m^−2^ increased from 1.4 to 30 min [172]. Nevertheless, the passivation layers also reduced current density, illustrating the tradeoff between defect suppression and carrier transport.

Among the available approaches, molecular passivation generally provides the most immediate improvement in radiative efficiency because it directly suppresses trap-assisted recombination. Precursor additives can treat the film interior and regulate crystallization, whereas post-deposition and bilateral treatments more selectively address surface and interfacial defects. However, strongly binding or bulky passivators may obstruct carrier transport, increase operating voltage, or alter the phase distribution, particularly in quasi-2D perovskites. Bilateral passivation therefore provides more complete defect control but requires careful optimization of interlayer thickness and conductivity. For large-area manufacturing, solution-compatible additives are attractive, although their concentration and processing window must remain sufficiently tolerant to variations in coating and drying conditions.

### 3.4. Transport Layers and Interface Engineering

A high-quality perovskite film alone does not guarantee an efficient PeLED. Electrons and holes must also be injected, transported, and confined within the emitting layer. Transport layers therefore control the injection barriers, carrier balance, recombination-zone position, operating voltage, and interfacial stability [173,174,175]. A typical device contains a transparent electrode, hole-transport layer (HTL), perovskite emitter, electron-transport layer (ETL), and metal electrode, with the transport-layer order reversed in an inverted architecture. Ideally, the HTL injects holes while blocking electrons, whereas the ETL performs the opposite function. Small energy barriers facilitate carrier injection, while sufficiently large barriers for the opposite carriers limit leakage. However, energy levels measured from isolated materials may not represent the operating interface because chemical reactions, surface dipoles, trapped charge, and mobile ions can alter the effective work function and internal electric field. Poor alignment increases operating voltage and promotes charge accumulation, trap-assisted recombination, ion migration, and degradation. Interfacial energy alignment should therefore be characterized for the relevant layer combination rather than estimated only from literature values.

PEDOT is widely used as a hole-injection layer because it is transparent, conductive, solution processable, and compatible with ITO [176,177]. Nevertheless, its acidic and hygroscopic nature may accelerate degradation, and its energy level is not ideal for every perovskite composition. Modification with salts, polymers, solvents, or interfacial dipoles can adjust its conductivity, work function, acidity, and surface properties. For example, CsCl-modified PEDOT improved hole injection and changed the phase distribution of a quasi-2D blue perovskite, producing 486-nm emission with a peak EQE of 16.07% [124]. This illustrates how transport-layer modification can simultaneously affect electrical injection and perovskite formation at the buried interface. Poly-TPD, TFB, and PVK can provide stronger carrier confinement but may increase the operating voltage because of their relatively low conductivity [178,179,180]. Their use in multilayer solution-processed devices also requires careful selection of orthogonal solvents. Inorganic HTLs such as NiO_x_ offer greater thermal and environmental stability, but their work function and conductivity depend strongly on stoichiometry and processing conditions. Reactive surface sites may also quench perovskite emission, requiring doping, surface passivation, or an ultrathin organic interlayer.

Organic ETLs such as TPBi provide electron transport and effective hole blocking, although they commonly require vacuum deposition and may be vulnerable to interfacial ion redistribution during electrical operation, although direct PeLED evidence is required to assign this transport-layer mechanism [181,182,183]. ZnO is an attractive alternative because of its high electron mobility, transparency, and solution processability [184,185]. However, its basic and defect-rich surface can react with organic cations or passivating ligands. In FAPbI_3_ PeLEDs, deprotonation of excess organic cations by ZnO promoted conversion of precursor intermediates into emissive perovskite [186,187,188]. Combined with defect passivation, this interaction produced a peak EQE of 19.6% [189], demonstrating that chemical compatibility can be as important as nominal energy-level alignment. Deposition of an upper transport layer may also damage the perovskite through solvent exposure, ligand removal, plasma treatment, sputtering, or atomic-layer deposition (ALD). Direct ALD growth of ZnO caused interfacial quenching in FAPbBr_3_ films, whereas inserting a solution-processed ZnO layer before ALD preserved a photoluminescence quantum yield of 71%. The resulting PeLEDs exhibited a turn-on voltage of 1.9 V and luminance above 300,000 cd m^−2^ [190].

Balanced injection is ultimately more important than maximizing the conductivity of either transport layer independently. Excess electrons or holes accumulate near an interface, increasing nonradiative recombination and electrochemical degradation. The recombination zone should instead remain inside the perovskite and away from quenching contacts. Its position can be adjusted through transport-layer mobility, thickness, doping, and injection barriers. In quasi-2D blue PeLEDs, changing the PEDOT thickness altered hole injection and shifted the recombination zone, resulting in a substantial change in EQE [191]. Effective interface engineering must therefore consider charge injection and chemical stability together. Its success should be evaluated through operating voltage, efficiency roll-off, voltage drift, and operational lifetime rather than peak EQE alone.

Thus, interface engineering is more effective when chemical stability and charge injection are considered together rather than through energy-level alignment alone. Organic transport layers are generally compatible with low-temperature solution processing but may permit ion penetration or degrade under heat, whereas dense inorganic layers can provide greater chemical and thermal stability but may damage the perovskite during deposition. Ultrathin interlayers and interface dipoles offer a useful compromise by reducing injection barriers, blocking ionic movement, and moving the recombination zone away from reactive contacts. Their effectiveness should be evaluated through operating voltage, efficiency roll-off, voltage drift, and lifetime rather than peak EQE alone.

### 3.5. Charge Balance and Optical Outcoupling

The external quantum efficiency of a PeLED can be understood as the product of three main factors: the efficiency of electron and hole injection, the probability that the injected carriers recombine radiatively, and the fraction of generated photons that escape from the device. Material passivation primarily improves radiative efficiency, whereas transport-layer engineering controls carrier injection. Even when both are highly optimized, a large fraction of the generated light can remain trapped inside a conventional planar device. High-performance PeLEDs must therefore combine balanced electrical operation with effective optical outcoupling.

Charge balance describes how closely the numbers of electrons and holes entering the emitting layer match each other [192,193]: when injection is balanced, most injected carriers can form electron–hole pairs and contribute to light emission; when one carrier dominates, the excess carriers accumulate at grain boundaries or interfaces, pass through the device as leakage current, or recombine through nonradiative pathways. The imbalance therefore lowers EQE even if the perovskite film has a high photoluminescence quantum yield. The balance is controlled by the injection barriers, carrier mobilities, and thicknesses of the transport and emitting layers. It can be adjusted through transport-layer selection, molecular doping, interface dipoles, blocking layers, or changes in layer thickness [194,195,196]. The objective is not necessarily to maximize both electron and hole currents, but to make their effective injection rates comparable. Reducing the injection barrier for an already dominant carrier can increase current density without producing a corresponding increase in luminance.

The recombination-zone position is closely related to charge balance [197,198,199]. If holes are transported more efficiently than electrons, recombination shifts toward the ETL/perovskite interface. If electron injection dominates, it shifts toward the HTL/perovskite interface. Both interfaces may contain traps or materials that quench excited states. A centered and sufficiently broad recombination zone generally reduces interfacial quenching and local carrier accumulation. However, its ideal position also depends on the optical field within the multilayer device because photons generated too close to the metal electrode are more likely to be lost through plasmonic coupling. Charge balance changes with current density: at low current density, injected carriers may first fill traps, resulting in weak electroluminescence and a low EQE. As the traps become occupied, radiative recombination increases and the EQE reaches a maximum. At still higher current density, the EQE commonly decreases because of Auger recombination, exciton–charge interactions, interfacial accumulation, Joule heating, and field-induced ion migration. This reduction is known as efficiency roll-off [200]. Efficiency measured only at the peak value can therefore overestimate practical performance. Display and lighting applications require the device to maintain efficiency at useful brightness. Accelerating radiative recombination can reduce the time during which carriers remain exposed to traps and competing nonradiative pathways. Similarly, interface and defect control have enabled near-infrared PeLEDs to retain EQEs above 10% at current densities approaching 1000 mA cm^−2^ [201].

Charge-only devices provide an approximate comparison of electron and hole transport because the electric field, traps, and ionic distribution can differ in a complete LED. Transient electroluminescence, capacitance, impedance spectroscopy, and spatial emission analysis can better reveal carrier accumulation, recombination-zone movement, and ion-induced changes in injection barriers. Even with perfect internal conversion, only about 20–30% of the generated light typically escapes a planar PeLED because of total internal reflection, waveguide modes, electrode absorption, and plasmonic losses. Outcoupling can be improved externally using lenses, roughened substrates, scattering films, or index-matching layers, or internally using nanostructures, optical spacers, low-index layers, and patterned emitters. For example, a hemispherical lens increased the EQE of a blue quasi-2D PeLED from 22.5% to 41.8% [202], while nanophotonic integration improved a MAPbBr3 device from 8.19% to 17.5% [203]. However, these structures require careful optical simulation and design because they can add manufacturing complexity or cause roughness, pinholes, leakage, and local electric-field concentration.

Cao et al. demonstrated that the emitting-layer morphology can function as an internal outcoupling structure without requiring an additional patterning process [156]. A precursor containing 5-aminovaleric acid (5AVA), FAI, and PbI_2_ was deposited on ZnO-PEIE and annealed to produce randomly distributed FAPbI_3_ platelets approximately 100–500 nm wide. Tomographic imaging and elemental mapping confirmed that an approximately 8 nm carbon-rich organic layer filled the gaps between the platelets rather than forming continuous layers above or below them. These low-refractive-index organic gaps, together with the corrugated TFB and metal-electrode layers, redirected photons from trapped waveguide modes into extractable directions. Meanwhile, 5AVA passivated surface defects, and the insulating material between platelets suppressed electrical leakage. Optical simulations estimated that the structured morphology increased the outcoupling efficiency from 21.8% for a continuous planar emitter to approximately 30%. The resulting PeLED achieved a peak EQE of 20.7% and retained wavelength- and viewing-angle-independent electroluminescence, demonstrating how morphology can improve both radiative efficiency and light extraction.

Oriented discrete crystallites have similarly produced estimated outcoupling efficiencies near 30% [204]. The morphology must remain electrically continuous enough to avoid leakage despite its optical texture. The refractive indices of the transport layers also influence optical confinement. A high-index ETL can support waveguide modes and increase coupling to the metal electrode. An anisotropic ETL with a low refractive index in the direction perpendicular to the film has been used to reduce waveguide and plasmonic losses while maintaining high electron mobility. This combined optical and electrical design enabled a green PeLED with an EQE of 32.1% [205]. Transport layers should therefore be selected using both their electronic properties and optical constants.

The orientation of the emitting transition dipoles provides another route to higher outcoupling [206,207]. Vertically oriented dipoles couple strongly to waveguide and surface-plasmon modes, whereas dipoles aligned parallel to the substrate emit a larger fraction of their energy into extractable modes. Most polycrystalline perovskite films contain approximately random dipole orientations, but layered perovskites and nanoplatelets can develop preferential orientation through controlled crystallization. In situ formation of oriented perovskite nanosheets has been used to tailor the optical dipoles of pure-red PeLEDs and raise the EQE above 30% without relying only on external extraction structures [208]. Orientation engineering is promising because it can improve extraction without necessarily changing the device footprint, but it requires reproducible control over crystal orientation across large areas.

Microcavity structures can also redistribute emission toward selected wavelengths and viewing directions [209,210,211]. By controlling the thicknesses of the transport and emitting layers and the reflectivity of the electrodes, constructive interference can increase forward emission. More complex photonic structures, including Tamm-plasmon configurations, can narrow both the spectral and angular distributions. However, strong cavity effects may create color changes with viewing angle, which are undesirable for general displays. They may be more useful for directional light sources, optical communication, or near-eye displays.

Consequently, charge balance should be established before optical outcoupling is optimized because enhanced extraction cannot compensate for severe leakage or interfacial recombination. Nanostructured emitting layers can produce large optical gains, but their rough surfaces may complicate multilayer deposition and large-area fabrication. Controlling the orientation of emitting dipoles is more compatible with planar device architectures, although it depends strongly on emitter dimensionality and molecular organization. The most practical designs will combine balanced injection and favorable optical modes without introducing additional processing steps that reduce film uniformity or operational stability.

## 4. Stability and Degradation

**Figure 6:**
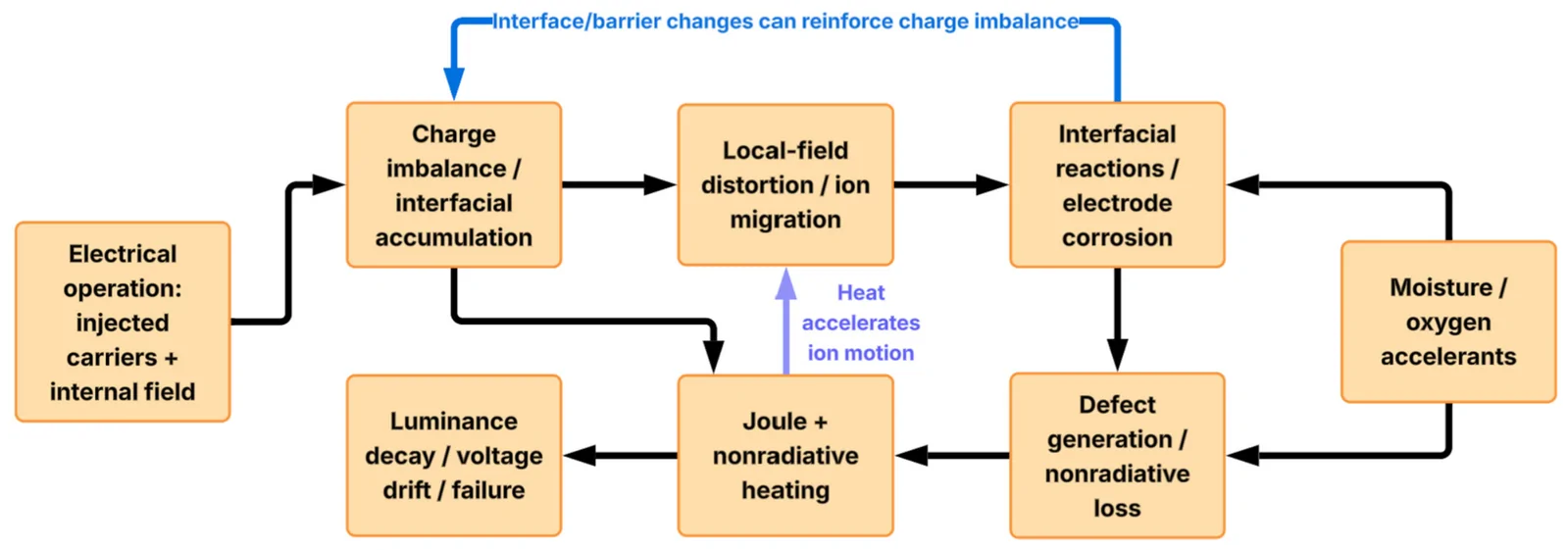
Coupled degradation pathways during PeLED electrical operation. Charge imbalance and interfacial accumulation distort local fields and promote ionic redistribution; ion motion and accumulated charge intensify interfacial reactions; defect generation and nonradiative/resistive losses increase heat generation; and elevated temperature further accelerates ion motion and chemical reactions. Moisture and oxygen act as external accelerants. The graphical elements in this conceptual schematic were generated using generative AI (ChatGPT 5.6–Sol) and subsequently reviewed and revised by the authors.

Operational stability remains the most important obstacle separating high-efficiency PeLED demonstrations from practical devices. During operation, the emitting layer is simultaneously exposed to injected charges, a strong electric field, Joule heating, mobile ions, and reactive interfaces. Moisture and oxygen may also enter through imperfect encapsulation or remain trapped inside the device during fabrication. These stresses reinforce one another, so degradation of an operating PeLED cannot usually be attributed to a single mechanism: charge imbalance promotes interfacial accumulation and local-field distortion, ionic redistribution modifies injection barriers, resistive and nonradiative losses generate heat, and elevated temperature accelerates ion motion and interfacial reactions (Figure 6). A useful stability analysis should distinguish reversible changes from permanent degradation. Ion redistribution may initially change the injection barriers and temporarily increase or decrease luminance. When the bias is removed, part of this change may recover. Prolonged operation, however, can produce irreversible chemical reactions, phase decomposition, electrode corrosion, and mechanical damage. The discussion below therefore focuses on degradation under electrical operation rather than only on the shelf stability of perovskite films.

### 4.1. Moisture, Oxygen, Heat, and Electrical Stress

Moisture can enter a PeLED through the edges of the package, microscopic defects in the encapsulation, or permeable organic transport layers [212,213,214]. Residual water may also be introduced during solution processing. Water molecules interact with the ionic perovskite lattice and can promote the formation of hydrated intermediate phases, increase ionic mobility, and eventually cause decomposition. In an operating device, the electric field and accumulated charges accelerate these processes. Moisture degradation may therefore remain limited during storage but become more severe after the device is electrically driven.

The effects of moisture are concentrated at defects, grain boundaries, and interfaces because these regions provide rapid diffusion pathways and chemically active sites. Current crowding near a pinhole or rough interface can produce localized heating and charge accumulation, making that region more vulnerable to moisture-induced degradation. The resulting defects increase nonradiative recombination and generate additional heat, creating a positive feedback loop that leads to the formation of dark spots and device failure. Oxygen is also more damaging under excitation than under dark storage. Electrons transferred to molecular oxygen can form reactive superoxide species, particularly at halide vacancies and undercoordinated metal sites. These species attack organic cations and the metal-halide framework. Reduced-dimensional perovskites and nanocrystals can be especially vulnerable because carriers can diffuse to exposed crystal edges with high defect densities. Edge-initiated photooxidation has been observed in reduced-dimensional emitters, while phosphine-oxide passivation of undercoordinated lead sites substantially improved operational stability [215]. Encapsulation can limit the continued entry of moisture and oxygen, but it cannot remove species already trapped within the device. Oxygen physically adsorbed during film preparation or transfer may remain at grain boundaries and interfaces after encapsulation. In addition, an encapsulation layer that is effective during room-temperature storage may become less effective when the operating device heats and expands. Encapsulation should therefore be combined with low-defect film processing and controlled-atmosphere fabrication.

Heat, on the other hand, is generated by resistive losses, nonradiative recombination, leakage current, and interfacial barriers [216,217]. The temperature rise may be much greater at local current-crowding sites than the average device temperature. Heat accelerates ion migration, ligand desorption, phase redistribution, and interfacial reactions. It may also soften organic transport layers, promote the loss of volatile organic species, and change the conductivity of the device stack. Because these processes increase resistance and nonradiative recombination, thermal degradation can become self-accelerating.

The relationship between efficiency and stability is therefore important. A device with poor charge balance requires more current and voltage to produce a given luminance, generating more heat. Efficient conversion of electrical power into light reduces this thermal load. FAPbI_3_ PeLEDs with improved energy-conversion efficiency showed greater operational stability, and their lifetime decreased strongly as the testing temperature increased from 25 to 45 °C [218]. High EQE alone, however, does not guarantee low heating because operating voltage, leakage current, and series resistance also determine electrical power loss.

Electrical stress directly affects both the electronic and ionic components of the device [219,220]. Under forward bias, injected electrons and holes may accumulate when their transport rates are unbalanced. Trapped charges can distort the local lattice, activate defects, and change the electric field. High voltage can also induce irreversible morphological and compositional changes in the perovskite layer. At moderate bias and short operating time, ion redistribution may temporarily improve injection and increase luminance. At higher bias or longer times, the same process accelerates material and interface degradation [221]. Electrical stress may first appear as luminance overshoot, voltage drift, efficiency hysteresis, or a change in the electroluminescence spectrum. Luminance overshoot occurs when ionic redistribution initially improves charge injection before degradation becomes dominant. It should not be interpreted as device stabilization. In 3D/2D hybrid PeLEDs, suppressing ion migration reduced the luminance-overshoot ratio from approximately 150% to 7.4% and extended operational lifetime by a factor of 21 relative to the 3D control devices [222]. Monitoring only the time required to reach half of the maximum luminance may hide this early transient behavior; both the initial and peak luminance should be reported. Operating conditions strongly influence the measured lifetime. Constant-current testing maintains the carrier-injection rate as the device resistance changes, but it may require an increasing voltage and accelerate late-stage degradation. Constant-voltage testing allows the current and luminance to decline together and may produce a different apparent lifetime. Pulsed driving reduces average heat generation and allows partial ionic relaxation between pulses, but results obtained under pulsed operation cannot be directly compared with continuous direct-current lifetimes. Current density, initial luminance, duty cycle, pulse width, temperature, atmosphere, and encapsulation must therefore be stated.

### 4.2. Ion Migration and Interfacial Degradation

Metal-halide perovskites conduct both electronic and ionic charge [223,224]. Halide ions and vacancies are generally the most mobile species, although organic cations and metal-related defects may also migrate. Under an applied electric field, positively and negatively charged ionic species move in opposite directions and accumulate at the interfaces. Grain boundaries, surfaces, and low-dimensional phase boundaries provide relatively low-energy migration pathways. Ion accumulation modifies the local electrostatic potential and injection barriers. In the early stages of operation, this redistribution may improve carrier injection, explain current–voltage hysteresis, or cause luminance overshoot. As ionic accumulation continues, it can screen the internal electric field, shift the recombination zone, and produce severe charge imbalance. These effects may be partially reversible when the bias is removed, especially before chemical changes occur. Ion migration can also change the perovskite composition. In mixed-halide emitters, field-driven halide redistribution creates regions with different bandgaps. Carriers preferentially recombine in the lower-bandgap regions, producing an emission shift and loss of color purity. In quasi-2D perovskites, ion movement can alter the distribution of quantum-well phases or promote phase disproportionation. In iodide-rich 3D emitters, vacancy formation and iodide redistribution may facilitate the formation of PbI_2_ and non-emissive structural phases. Once ions leave the perovskite and enter a transport layer or electrode, degradation becomes more difficult to reverse. Halide ions can react with metal electrodes such as Al or Ag, producing insulating metal-halide compounds and electrode corrosion. Metal atoms can also diffuse toward the perovskite and create deep traps. Organic cations or volatile decomposition products may enter the adjacent layers, while acidic or basic transport-layer surfaces may initiate additional reactions. Recent operando electron-microscopy observations have shown that PeLED failure is strongly localized at buried interfaces. Bias-induced halide redistribution, lattice strain, phase transformation, and Al-electrode corrosion developed together, indicating that device failure can be governed by coupled structural and electrochemical reactions rather than degradation of the emitting states alone [225]. This evidence highlights the need to treat the PeLED as a complete multilayer system.

The interfaces also concentrate mechanical stress [226]. Different layers have different thermal-expansion coefficients and may expand unevenly as the device heats. Repeated operation and cooling can produce interfacial strain, void formation, or partial delamination. These mechanical changes create additional pathways for ion movement and oxygen or moisture penetration. Rough surfaces and incomplete layer coverage further concentrate the electric field and increase local current density. Interfacial degradation is closely connected to carrier imbalance [227]. When excess electrons or holes accumulate near a transport layer, they can participate in redox reactions involving halides, metal ions, or electrode atoms. Moving the recombination zone away from reactive interfaces and reducing excess carrier accumulation can therefore improve stability even without changing the intrinsic perovskite composition. This is one reason why defect passivation, charge balance, and interface engineering must be optimized together.

### 4.3. Strategies for Improving Operational Lifetime

Improving operational lifetime requires several complementary strategies. A moisture barrier alone cannot stop field-driven ion migration, while suppressing ion migration will not protect an unencapsulated device from environmental exposure. The most effective designs stabilize the perovskite lattice, reduce defect-assisted ionic motion, protect the interfaces, balance charge injection, limit heating, and prevent external species from entering the device.

The first strategy is to reduce the density of defects that act as both nonradiative recombination centers and ion-migration pathways [228,229]. Strongly binding passivators can coordinate with undercoordinated metal ions and fill halide vacancies. Molecules that interact with both cations and anions can stabilize the ionic lattice more effectively than single-site passivators. A dipolar molecular stabilizer introduced at FAPbI_3_ grain boundaries suppressed ion migration and the formation of PbI_2_, producing near-infrared PeLEDs with extrapolated T_50_ lifetimes of 11,539 h at approximately 5 mA cm^−2^ and 32,675 h at approximately 3.2 mA cm^−2^ [230]. These extrapolated values illustrate the potential of molecular stabilization, but they should not be compared directly with lifetimes measured at a specified initial luminance or current density. Surface ligands can also create a steric barrier to ionic rearrangement. Phenylalkylammonium molecules with stronger binding and suitable chain length suppressed iodide migration while passivating surface defects. The optimized devices reached a directly measured T_50_ of 130 h under constant-current operation at 100 mA cm^−2^. Excessively long or insulating ligands must be avoided because they increase operating voltage and heat generation.

Dimensionality engineering can restrict ion movement and improve structural stability. Incorporating a controlled amount of 2D perovskite into a 3D matrix can stabilize grain boundaries and reduce ionic reorientation. However, an uncontrolled distribution of low-*n* phases can introduce defects and transport barriers. Selective removal of undesirable defect-rich phases using a solvent-sieving method produced quasi-2D PeLEDs with an EQE of 29.5% and a measured T_50_ of 18.67 h at an initial luminance of 12,000 cd m^−2^ [61]. The estimated lifetime at lower luminance was much longer, but such extrapolations should be distinguished from directly measured values. Kim et al. combined the strong carrier confinement of nanocrystals with the favorable transport properties of three-dimensional perovskites through an in situ core/shell treatment [129]. Benzylphosphonic acid (BPA) initially bound to defective surfaces, while a subsequent 30 s BPA treatment penetrated and divided the large mixed-cation bromide crystals into approximately 10 nm cores surrounded by BPA. The new P–O–Pb component in the O 1s spectra confirmed the formation of covalent Pb–O–P bonds, which passivated undercoordinated Pb atoms and bromide-vacancy sites. The smaller cores strengthened carrier confinement, while the chemically bound BPA shell reduced trap-assisted recombination without severely restricting charge transport. The resulting green PeLEDs achieved a peak EQE of 28.9%, an average EQE of 25.2 ± 1.6% across 40 devices, and a maximum luminance of approximately 470,000 cd m^−2^. More importantly, they exhibited a measured T_50_ of 520 h at 1000 cd m^−2^; the lifetime at 100 cd m^−2^ was estimated, rather than directly measured, to exceed 30,000 h.

The second strategy is to stabilize both interfaces of the emitting layer [231]. Ultrathin passivation or blocking layers can reduce surface traps, prevent direct reactions with transport layers, and restrict halide movement toward the electrodes. The barrier should be continuous but thin enough to permit carrier tunneling or transport. Bilateral interface treatment is often more effective than passivating only the exposed upper surface because degradation can begin at either the top or buried interface. Robust transport layers and electrodes are also needed. Chemically inert layers can prevent reactive contact with the perovskite, while dense inorganic layers can reduce the diffusion of ions and metal atoms. However, deposition methods such as sputtering or atomic-layer deposition may damage the perovskite. Protective intermediate layers can allow robust inorganic transport layers to be added without direct exposure. A bilayer ZnO electron-injection structure protected the FAPbBr_3_ surface during ALD processing and enabled a measured T_50_ of 350 h at 1000 cd m^−2^, corresponding to an estimated 17,000 h at 100 cd m^−2^ [190].

The third strategy is to reduce electrical and thermal stress. Studies of thermal and electrical stress in related OLED systems provide useful transferable context, although their results should not be interpreted as direct evidence for PeLED thermal-stress mitigation [232]. Balanced electron and hole injection minimizes excess interfacial charge and lowers the current required to obtain a given luminance. Reducing series resistance and injection barriers lowers the operating voltage and Joule heating. Improved heat spreading may reduce local temperature rise, although the magnitude of this benefit depends on device architecture, layer thermal properties, and operating current density. Thermal management becomes especially important for high-brightness lighting, active-matrix displays, and electrically pumped laser applications. Driving conditions can also be optimized. Pulsed operation can reduce average heating and may delay ionic accumulation, while carefully selected duty cycles can maintain brightness without continuous high-current stress. However, pulsed driving does not eliminate the underlying degradation mechanisms, and very high peak currents may still cause Auger recombination or local damage. Practical lifetime measurements should therefore include operating modes representative of the intended application.

Finally, encapsulation remains necessary [233,234]. Thin-film barriers, glass lids, edge seals, and getter materials can prevent moisture and oxygen ingress. Effective encapsulation should also tolerate thermal cycling and block the escape of volatile perovskite decomposition products. For flexible PeLEDs, the barrier must maintain its integrity during bending. Encapsulation results should be reported together with the barrier materials and sealing method because different packages can produce substantially different device lifetimes.

The coupled feedback among charge accumulation, ionic redistribution, heating, and interface reactions is summarized in Figure 6. Charge accumulation can distort local fields and promote ion migration, while migrating ions can alter injection barriers and accelerate interfacial reactions. Defect formation and nonradiative or resistive losses increase local heating, which can further accelerate ionic motion and chemical degradation; moisture and oxygen act as external accelerants.

Overall, the available lifetime results show that molecular stabilization, dimensionality control, core/shell formation, and interface protection can all delay PeLED degradation, but their reported lifetimes are not directly interchangeable. The Ding et al. result is particularly relevant to high-brightness operation, whereas the core/shell and bilayer-ZnO devices provide stronger measured lifetimes at 1000 cd m^−2^. By contrast, the longest values reported for dipolar molecular stabilization were extrapolated at lower current densities. Future studies should therefore prioritize directly measured lifetime at application-relevant brightness, together with voltage drift, spectral stability, encapsulation conditions, active area, and device statistics. Table 3 consolidates the major case studies. Cross-study comparisons of lifetime versus initial luminance and peak EQE versus electrically active area are presented later in Section 5. These summaries reveal broad trends but do not establish a universal scaling law because architectures, colors, encapsulation, driving modes, and test conditions differ.

Lifetime extrapolation should be distinguished explicitly from direct measurement [129]. A commonly used acceleration-factor relationship has the form L_0_^n^ × T_50_ = constant, where L_0_ is the initial luminance and n is fitted empirically. Extrapolation is most defensible when n is obtained from multiple directly measured T_50_ values for the same device architecture over a range of initial luminances, rather than assumed from another material system or device. In the core/shell nanocrystal study summarized here, the exponent was fitted as n ≈ 1.68 using measurements from 21 devices rather than assumed from a generic OLED value. Reported extrapolated lifetimes should therefore be accompanied by the measured data used for the fit, the fitted exponent, driving mode, encapsulation condition, and temperature.

## 5. Emerging Applications

Most PeLED research has focused on improving efficiency, brightness, and lifetime in conventional planar devices. However, the low-temperature processing, color tunability, mechanical compatibility, and mixed ionic-electronic behavior of perovskites also enable device functions that are difficult to obtain from conventional LEDs. Important emerging directions include directly patterned displays, reconfigurable emission, flexible and transparent light sources, optical communication, and devices capable of performing more than one optoelectronic function.

### 5.1. Patterned and Reconfigurable Displays

Practical displays require individually addressable red, green, and blue pixels with small dimensions, uniform brightness, low electrical crosstalk, and compatibility with a transistor backplane. Conventional PeLEDs are generally fabricated as continuous films, so additional patterning is needed to define pixels. Potential methods include inkjet printing, transfer printing, shadow-mask deposition, photolithography, nanoimprinting, and spatially controlled crystallization. Patterning perovskites is difficult because their ionic lattices are easily damaged by polar solvents, photoresists, developers, plasma exposure, heat, and mechanical pressure [235,236]. Nanocrystal emitters may additionally lose surface ligands during patterning, leading to aggregation and reduced photoluminescence. In full-color displays, the solvents used to deposit one color must not dissolve or chemically alter previously deposited pixels. A successful process must therefore provide high spatial resolution without reducing the efficiency, color purity, or lifetime of the original emitting material. Inkjet printing is attractive because it is mask-free and can place different emitters at selected locations [237]. However, droplet spreading, coffee-ring formation, nozzle variation, and solvent evaporation can produce nonuniform pixel thickness. Transfer printing avoids direct exposure of the perovskite to aggressive solvents, but incomplete transfer and mechanical damage may reduce yield. Vacuum deposition through fine masks provides better compatibility with existing OLED manufacturing, although mask deformation and material diffusion limit resolution over large areas.

**Figure 7:**
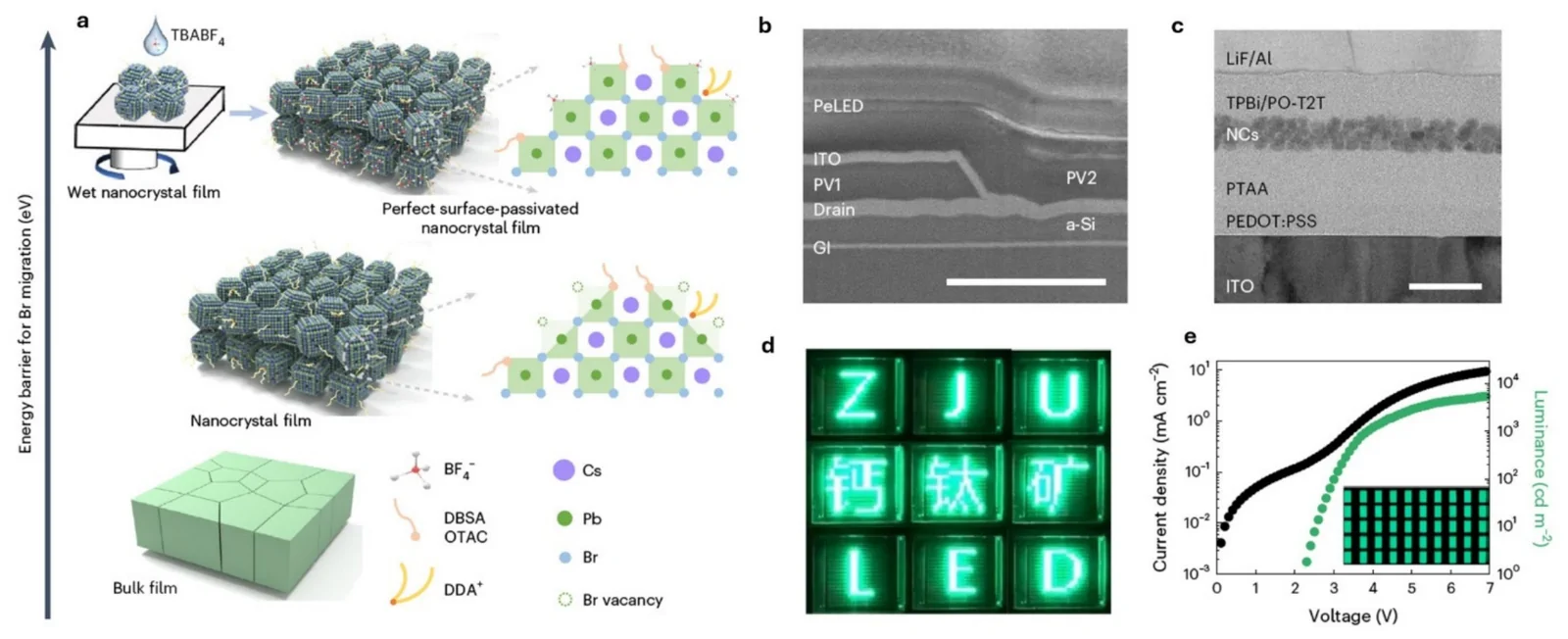
Individual-particle passivation and active-matrix integration of CsPbBr_3_ nanocrystal PeLEDs. (**a**) Schematic comparison of bulk, untreated nanocrystal, and TBABF_4_-treated nanocrystal films, showing how BF_4_^−^ passivation of surface Br vacancies increases the barrier to ion migration. (**b**) Cross-sectional SEM image of the PeLED integrated on an amorphous-silicon TFT backplane; scale bar, 1 μm. (**c**) Cross-sectional HRTEM image of the ITO/PEDOT:PSS/PTAA/CsPbBr_3_ nanocrystal/TPBi/PO-T2T/LiF/Al device; scale bar, 50 nm. (**d**) Photographs of independently addressed green pixels displaying representative patterns; the Chinese characters mean “perovskite” in English. (**e**) Current-density–luminance–voltage characteristics of an individual pixel; the inset shows uniform emission across the pixel array. Reproduced from Reference [42] under CC BY 4.0, with permission from Springer Nature.

To make nanocrystal PeLEDs suitable for active-matrix displays, Gao et al. introduced TBABF_4_ during the deposition of CsPbBr_3_ nanocrystal films [42]. The BF_4_^−^ ions passivated surface Br vacancies on individual nanocrystals, increasing the measured activation energy for ion migration from 190 to 478 meV and reducing the electroluminescence rise time from more than 20 ms in untreated devices to within 10 μs after treatment. Figure 7a illustrates this passivation mechanism, while Figure 7b,c show the integration of the treated emitter with an amorphous-silicon TFT backplane and appropriate charge-transport layers. The resulting 16 × 16, 30-ppi green active-matrix display produced uniformly addressable patterns and favorable current-density–luminance characteristics (Figure 7d,e). A representative pixel reached a peak EQE of 23.3% and maintained an EQE above 20% between 500 and 3000 cd m^−2^. The approach was also extended to 90-ppi red, green, and blue active-matrix displays with response times of tens of microseconds, demonstrating the importance of suppressing ion migration for high-refresh-rate PeLED operation.

**Figure 8:**
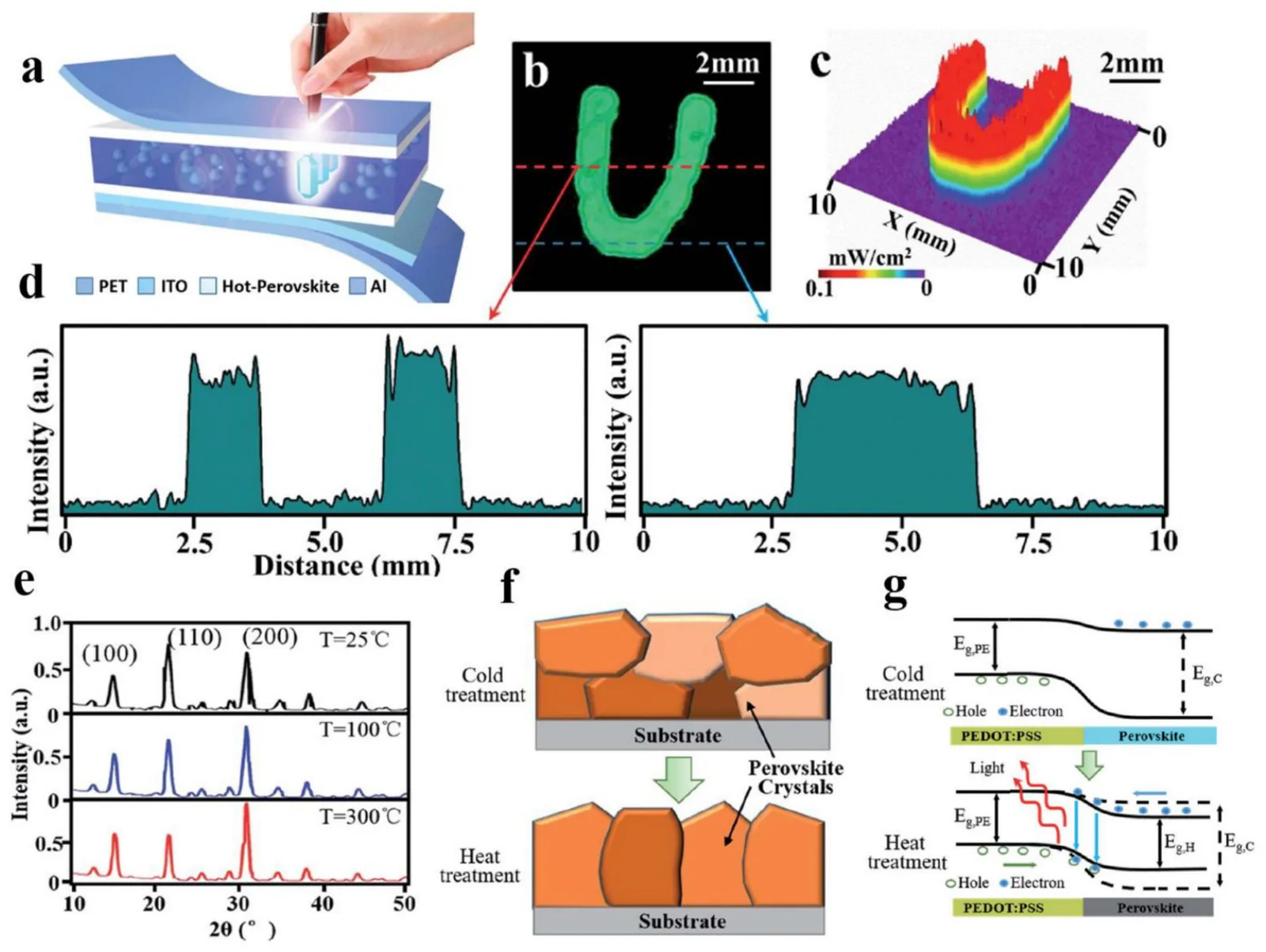
Thermal patterning and emission mechanism of a writable and wipeable PeLED. (**a**) Schematic of localized thermal writing on the flexible PET/ITO/PEDOT:PSS/CsPbBr_3_/LiF/Al device. (**b**) Electroluminescence image of a handwritten “U” and (**c**) its three-dimensional intensity distribution; scale bars, 2 mm. (**d**) Intensity profiles along the two marked lines in (**b**), showing relatively uniform emission. (**e**) XRD patterns of CsPbBr_3_ films treated at 25, 100, and 300 °C. (**f**) Schematic of the thermally induced improvement in crystal organization. (**g**) Proposed energy-level diagrams illustrating the enhanced charge injection and recombination following thermal treatment. Reproduced from Reference [239] under CC BY-NC 3.0, with permission from The Royal Society of Chemistry.

More recently, patterned perovskite quantum-dot superlattices were integrated onto a commercial thin-film-transistor backplane to produce a 1.85-inch active-matrix display with grayscale control and video playback [238]. These demonstrations represent important movement from isolated test pixels toward complete display systems. Bi et al. developed a flexible, thermally writable PeLED based on a PET/ITO/PEDOT:PSS/CsPbBr_3_/LiF/Al structure [239]. Following low-temperature treatment of the emitter, selected regions were locally heated with a pen at approximately 300 °C for less than 2 s, improving crystallinity and lowering their turn-on voltage. As shown in Figure 8a–d, this process produced a handwritten “U” with relatively uniform emission, while the surrounding untreated area remained dark at the same operating voltage. The XRD results showed temperature-dependent changes in the intensities of the (100), (110), and (200) reflections without corresponding peak shifts, indicating changes in crystallinity and crystal organization rather than a phase transformation (Figure 8e,f). The authors attributed the enhanced emission to this structural reorganization and a more favorable charge-recombination pathway at the PEDOT:PSS/CsPbBr_3_ interface (Figure 8g). Thermally treated and untreated regions differed in turn-on voltage by approximately 5 V, while the treated regions produced more than ten times greater electroluminescence intensity and reached a peak EQE of approximately 1.35% after treatment at 300 °C. The pattern could be removed by applying a voltage of approximately 11 V or higher. Joule heating then crystallized the previously untreated regions, causing the entire device to emit with similar intensity. This “wiping” process did not remove or reverse the locally treated pattern; instead, it eliminated the optical contrast by converting the surrounding area into the same highly emissive state. Importantly, the crystallization process was irreversible, so a wiped device could not subsequently be rewritten. The device is therefore more accurately described as thermally writable and electrically erasable once, rather than repeatedly rewritable. The study also demonstrated local handwriting recognition, thermal stamp printing, bending beyond 80°, and operation after repeated bending and on/off cycling. These results show how spatial control of crystallization can replace conventional material-removal patterning. Possible applications include temporary labels, signatures, anti-counterfeiting marks, one-time indicators, and human–machine interfaces.

### 5.2. Flexible and Transparent PeLEDs

Flexible PeLEDs could enable foldable displays, conformable lighting, wearable indicators, electronic skin, and light sources integrated onto curved surfaces [240,241]. Perovskites are well suited to these applications because they can be deposited at relatively low temperatures on plastic or metal-foil substrates. However, the complete device must remain electrically and optically stable during deformation. Mechanical failure often begins in the electrodes and interfaces rather than in the perovskite alone. Conventional ITO is transparent and conductive but is brittle when deposited on plastic. Repeated bending can create cracks that increase resistance and produce nonuniform current injection. Alternative transparent electrodes include silver nanowire networks, conductive polymers, metal meshes, graphene, ultrathin metals, and oxide-metal-oxide stacks. These electrodes must combine high optical transmission, low sheet resistance, smooth surfaces, and chemical compatibility with the transport layers. A rough nanowire electrode, for example, can puncture thin functional layers and create leakage unless it is planarized.

The perovskite emitting layer is also susceptible to cracking because it is crystalline and mechanically different from polymer substrates and organic transport layers [242,243]. Low-dimensional perovskites, nanocrystal films, quantum-wire arrays, and perovskite-polymer composites can provide better strain tolerance than dense brittle films. Polymers can bridge grain boundaries, distribute strain, and limit crack propagation, although excessive polymer content may reduce charge transport. Device geometry is another important consideration [244,245,246]. Placing the brittle functional layers close to the neutral mechanical plane reduces the strain they experience during bending. Thin substrates and ultrathin device stacks can achieve smaller bending radii, while barrier layers must remain continuous under repeated deformation. Mechanical testing should report bending radius, calculated strain, number of cycles, bending direction, and device performance during and after deformation. A photograph of a bent device alone does not establish mechanical durability.

Flexible full-color PeLEDs have been demonstrated using all-inorganic perovskite quantum wires grown within porous alumina templates [247,248,249]. The confined wire structure improved crystal quality, passivation, and light extraction while mechanically supporting the perovskite. Blue, sky-blue, green, and pure-red devices achieved peak EQEs of 12.41%, 16.49%, 26.09%, and 9.97%, respectively. Uniform emission was demonstrated from flexible devices with areas of 1.5 × 1.5 cm^2^ and larger rigid devices up to 3 × 3.5 cm^2^ [250]. Although these results demonstrate color and area scalability, long-term bending and operational lifetime remain important considerations. The writable and wipeable PeLED reported by Bi et al. also provides a useful flexible-device example because its CsPbBr_3_ emitting layer was integrated on an ITO-coated PET substrate [239]. The patterned devices retained electroluminescence under bending angles exceeding 80° and after repeated deformation. However, the brittle ITO electrode and high-temperature writing process may limit repeated or severe flexing in practical systems. More flexible transparent electrodes and lower-temperature patterning methods would therefore be needed to strengthen this concept.

Transparent PeLEDs address a different application need. Instead of merely bending, the entire device must transmit visible light while emitting in a selected spectral region. This requires transparent or semitransparent electrodes on both sides and transport layers with low absorption. Replacing a reflective metal cathode with an ultrathin metal or oxide-metal-oxide electrode increases transmission but can raise sheet resistance and reduce carrier injection. Optical interference in the multilayer stack also creates a tradeoff among transparency, emission intensity, and viewing-angle dependence. Transparent near-infrared PeLEDs are particularly attractive because they can be placed over a visible display without strongly affecting the displayed image. A transparent FAPbI_3_ PeLED using an Al/ITO/Ag/ITO top electrode achieved an average visible transmittance above 55%, an emission wavelength of 799 nm, an EQE of 5.7%, and a functional area of 120 mm^2^ [251]. Such devices could provide covert illumination for facial recognition, eye tracking, depth sensing, and augmented-reality systems while sharing the same surface area as a conventional display.

For both flexible and transparent PeLEDs, performance should be evaluated as a combined system rather than through EQE alone. Flexible devices require mechanical lifetime, stable electrode resistance, and barrier integrity. Transparent devices require average visible transmittance, color neutrality, haze, and emission efficiency from both viewing directions. Combining flexibility and transparency remains particularly challenging because the electrodes, encapsulation, and emitting layer must simultaneously satisfy electrical, optical, and mechanical requirements.

### 5.3. Optical Communication and Multifunctional Devices

PeLEDs are also being investigated as modulated light sources for visible and near-infrared communication [252]. Optical communication encodes data by rapidly switching the light source between different intensity levels. Perovskites possess short intrinsic radiative lifetimes and potentially fast emission, but the response speed of a complete PeLED is also limited by charge injection, device capacitance, traps, and ion migration. Ion migration is especially important because it changes the internal field and injection barriers over timescales ranging from microseconds to seconds. The resulting slow current and luminance transients reduce modulation bandwidth and introduce signal distortion. Suppressing ion migration and improving charge injection are therefore necessary for communication as well as display refresh rate.

Directly driven perovskite emitters have already demonstrated data transmission. A perovskite LED system using on-off keying achieved a data rate of 90 Mbit s^−1^, while experiments showed that halide migration changed emission intensity and communication performance [253]. This result demonstrates the feasibility of PeLED-based visible-light communication but also reveals that material stability and signal stability are closely linked. The microsecond-response red, green, and blue PeLEDs discussed above are also relevant to optical communication. Their faster response resulted from individual nanocrystal passivation that suppressed ion motion and improved crystallinity. However, PeLED modulation speeds remain below those of mature inorganic micro-LEDs and laser diodes. Further progress requires lower device capacitance, faster injection layers, reduced active area, higher output power, and stable operation at high modulation current.

Bao et al. demonstrated a dual-functional FAPbI_3_ perovskite diode that operated as an LED under forward bias and as a photodetector under zero or reverse bias, allowing identical devices to serve as optical transmitters and receivers [254]. The diode produced near-infrared emission at approximately 804 nm with a peak EQE of 21.2%, while also providing sub-picowatt detection and a specific detectivity above 2 × 10^12^ Jones. Transient measurements at 100 Hz and 100 kHz confirmed rapid switching, and reducing the device area from 7.25 to 0.10 mm^2^ increased the −3 dB emission bandwidth from 1.8 to 21.5 MHz by lowering parasitic capacitance. In both interchip and intrachip configurations, light from one diode generated a repeatable photocurrent in another identical diode. The full-area optical link had a cutoff frequency of approximately 1.2 MHz and supported intrachip digital transmission at 1 Mbit s^−1^. Two diodes were also integrated as the emitter and detector of a monolithic pulse sensor, demonstrating the potential of PeLEDs for compact communication and sensing systems. This dual operation can reduce the number of separate components in compact optical systems. A pixel could potentially emit information, detect a response, and switch between the two functions using the same active material. Possible applications include short-range chip-to-chip links, interactive displays, proximity sensing, wearable health monitors, and optical human–machine interfaces. Near-infrared PeLEDs are especially suitable for these applications because their emission is invisible to the human eye and overlaps with common photodetection and biometric-sensing wavelengths.

Multifunctionality can also arise from spatial or external control of emission [255]. Thermally written PeLED patterns can combine display and information-recording functions, while flexible devices can integrate visual indicators with strain, pressure, or motion sensing. Patterned devices may serve as anti-counterfeiting labels by producing a voltage-dependent optical response that is more difficult to reproduce than a printed image. However, such applications require clear evidence that the additional function is repeatable, stable, and distinguishable from ordinary device degradation.

The main challenges for communication and multifunctional PeLEDs are response speed, output power, operational lifetime, signal reproducibility, and system integration [256,257,258]. Ion migration may cause the modulation response to change during use, while high-frequency driving and high current density increase heat generation. Lead-containing devices also require effective containment, particularly in wearable applications. Future research should therefore evaluate these devices using application-level measures such as modulation bandwidth, bit-error rate, signal-to-noise ratio, power consumption, response stability, and performance after repeated mechanical or electrical cycling.

Therefore, emerging PeLED applications are moving beyond efficient light generation toward spatial programmability, mechanical integration, transparency, communication, and combined emission-detection functions. The most convincing demonstrations are those in which the new functionality is supported by a practical device architecture and quantified under realistic operating conditions. Table 4 indicates that active-matrix displays and patterned quantum-dot arrays currently provide the clearest system-level integration, whereas flexible, transparent, communication, and multifunctional concepts remain more strongly constrained by incomplete lifetime, scale-up, or integration evidence.

**Figure 9:**
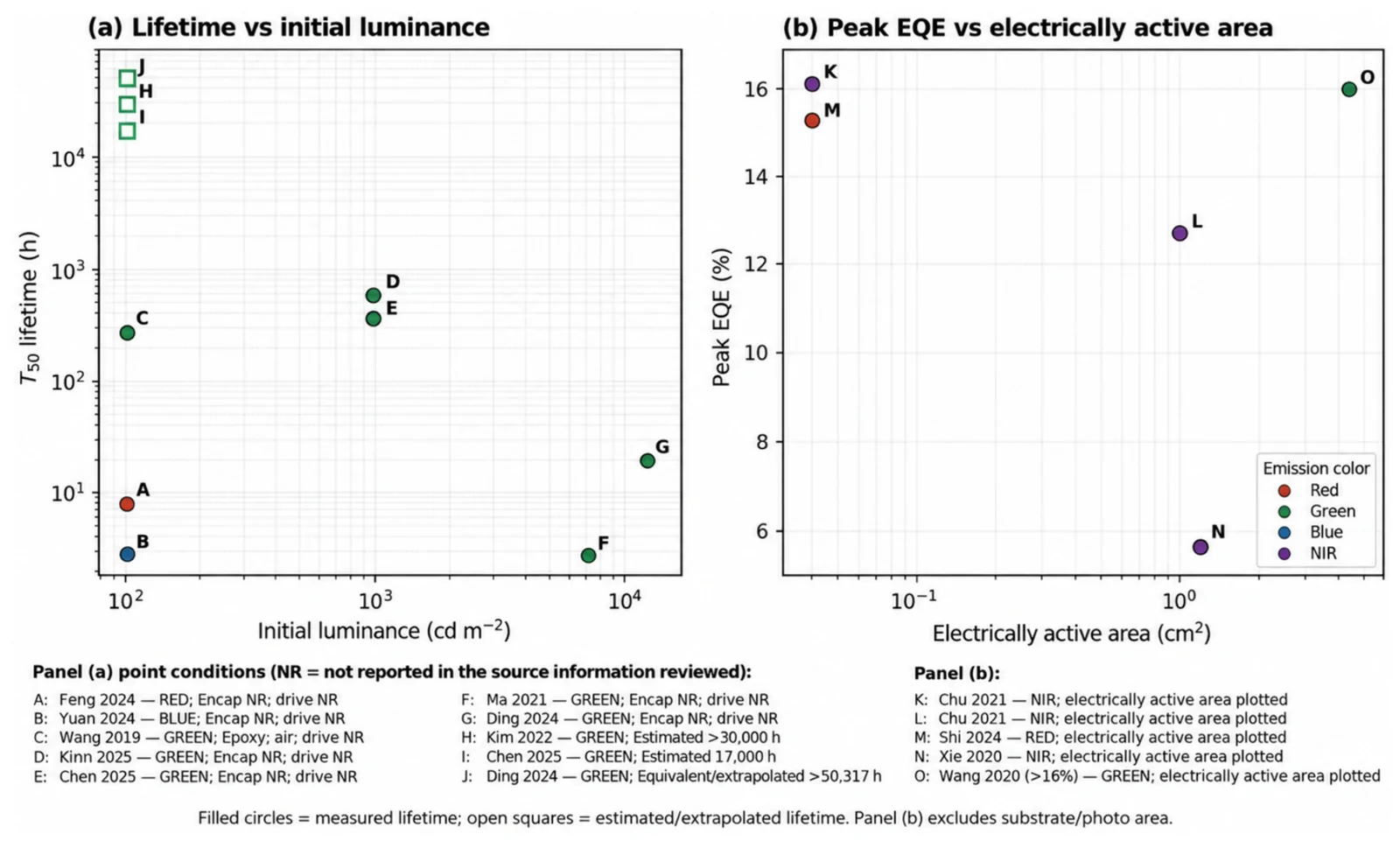
Quantitative cross-study summaries. (**a**) Reported T50 lifetime versus initial luminance, with directly measured values shown as filled circles and estimated/extrapolated values as open squares; emission color, encapsulation information, and driving information are stated explicitly where available, with NR used when not reported. (**b**) Peak EQE versus electrically active area; substrate or photograph area is not substituted for active area. These cross-study comparisons reveal broad trends but do not establish a universal scaling law because device architecture, emission color, encapsulation, and test conditions differ among studies. Figure prepared with generative-AI assistance using literature data compiled and verified by the authors.

Across these application classes, quantitative comparison among reported PeLEDs remains complicated by differences in operating and device conditions. Operational lifetime depends not only on the intrinsic stability of the emitting material, but also on initial luminance, current density, driving mode, encapsulation, device architecture, and thermal environment. Similarly, changes in device area can affect film uniformity, defect probability, current distribution, and series resistance, making peak EQE values from small-area devices difficult to compare directly with larger-area demonstrations. Consequently, cross-study trends are most useful when the relevant testing conditions and active areas are reported explicitly and when measured lifetimes are distinguished from extrapolated values. Figure 9 therefore provides two complementary comparisons: reported operational lifetime as a function of initial luminance and peak EQE as a function of electrically active area.

## 6. Conclusions and Outlook

PeLEDs have progressed from low-efficiency laboratory devices to highly competitive light emitters, with peak EQEs exceeding 30% and strong performance across red, green, and near-infrared wavelengths. This progress has resulted from coordinated advances in composition and dimensionality control, crystallization regulation, defect passivation, transport-layer design, charge balance, and optical outcoupling. The central lesson is that these strategies cannot be optimized independently. A treatment that improves photoluminescence may restrict charge transport, while faster injection may increase interfacial accumulation, ion migration, and degradation. Future development must therefore treat the PeLED as a complete electrical, optical, chemical, and thermal system rather than focusing only on the emitting film.

Operational stability remains the primary barrier to commercialization. Device failure arises from coupled processes involving defects, mobile ions, Joule heating, charge imbalance, interfacial reactions, and environmental exposure. Promising results from molecular stabilization, dimensionality control, core–shell structures, robust transport layers, and bilateral interface protection show that long-lived operation is achievable. However, lifetime comparisons remain difficult because studies use different initial luminance, current density, driving modes, device areas, and extrapolation methods. Future work should emphasize directly measured lifetimes at application-relevant brightness and report voltage drift, spectral evolution, encapsulation conditions, active area, and device-to-device statistics alongside T_50_.

Among the lifetime demonstrations discussed in this review, a directly measured T_50_ of 520 h at 1000 cd m^−2^ remains substantially shorter than the extrapolated value above 30,000 h at 100 cd m^−2^ reported for the same core/shell nanocrystal device. This contrast highlights how strongly apparent lifetime depends on operating brightness and extrapolation protocol, and why commercial readiness should be judged from directly measured, application-specific tests rather than extrapolated low-brightness values alone.

Efficient and stable blue emission is another critical priority. Although blue PeLEDs have achieved EQEs above 20%, deep-blue devices still suffer from mixed-halide phase segregation, uncontrolled quasi-2D phase distributions, inefficient charge transport, and rapid spectral drift. Progress will require compositions that maintain their bandgap under electrical bias, stronger control over quantum-well distribution, and interfaces that suppress ion accumulation without introducing large injection barriers. Efficiency should also be evaluated at practical luminance and together with color coordinates, spectral stability, roll-off, and lifetime.

Manufacturing challenges must receive equal attention. Record devices are generally fabricated over small areas using tightly controlled spin-coating processes, whereas commercial displays require uniform large-area deposition, high-resolution RGB patterning, low pixel-to-pixel variation, and integration with transistor backplanes. Printing, blade coating, transfer patterning, and vacuum deposition offer possible routes, but they must preserve film quality and passivation while increasing throughput and yield. Optical outcoupling structures must likewise be compatible with scalable fabrication and should not introduce roughness, leakage, or additional instability. Standardized testing across larger active areas and multiple fabrication batches will be essential for demonstrating reproducibility.

**Figure 10:**
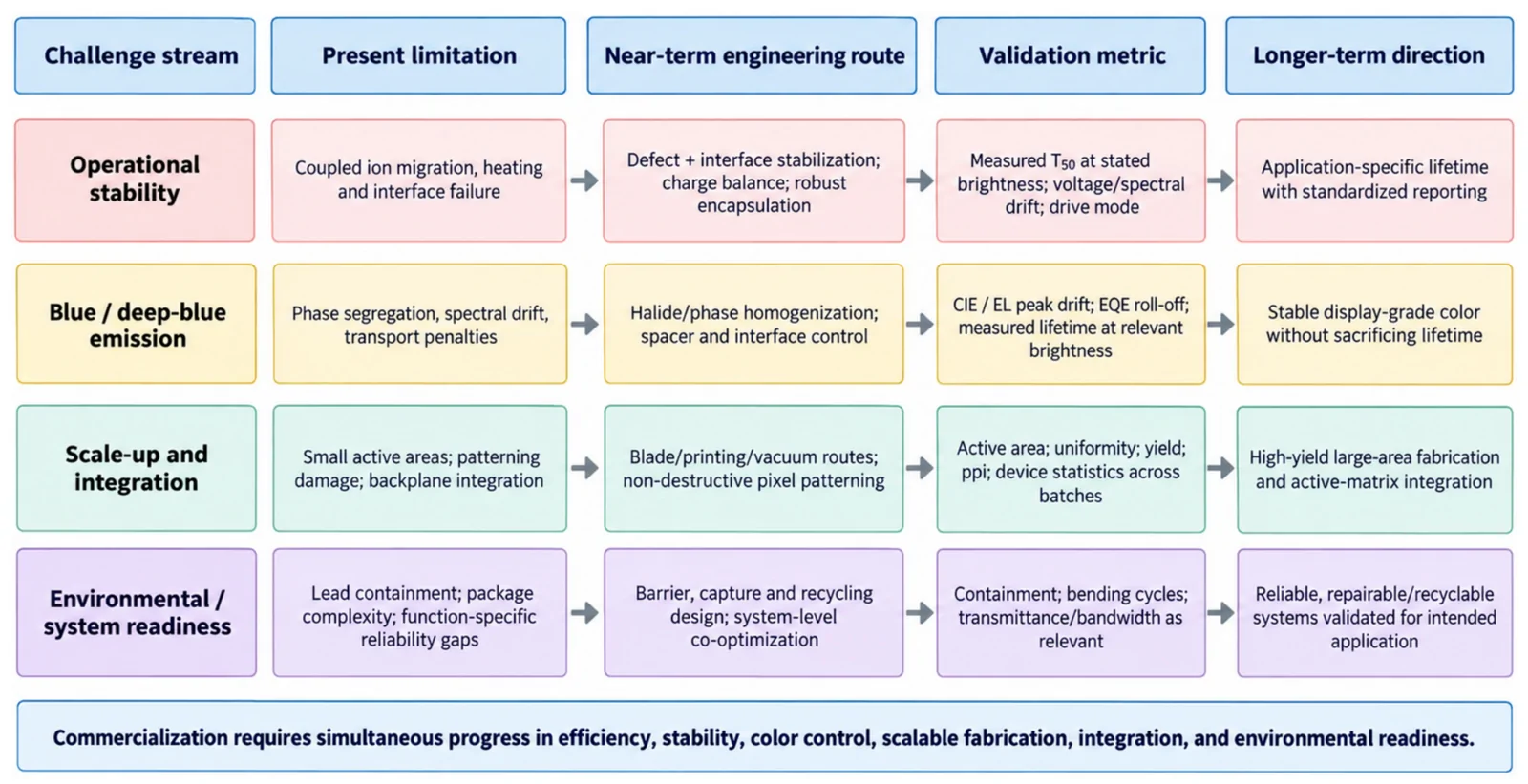
Commercialization roadmap for PeLEDs. Four coupled challenge streams are mapped from present limitations through near-term engineering routes and validation metrics to longer-term directions. The roadmap deliberately avoids a universal lifetime threshold and instead emphasizes application-specific, directly measured performance under defined operating conditions. Original roadmap created for this review. The graphical elements in this conceptual schematic were generated using generative AI (ChatGPT 5.6–Sol) and subsequently reviewed and revised by the authors.

Beyond conventional displays, PeLEDs offer distinctive opportunities in flexible and transparent electronics, optical communication, reconfigurable emission, and combined light-emission and detection systems. These applications should be evaluated using function-specific measures such as bending endurance, visible transmittance, modulation bandwidth, bit-error rate, and cycling stability rather than EQE alone. At the same time, lead containment, recycling, and end-of-life management must be incorporated into device and package design. The future of PeLED technology will depend less on further improvement of isolated efficiency records and more on combining high efficiency with stable color, long lifetime, scalable processing, environmental responsibility, and reliable system-level operation. The commercialization roadmap in Figure 10 emphasizes simultaneous progress in operational stability, stable blue emission, scalable fabrication and integration, and environmental or system-level readiness rather than a single record EQE.

## Figures and Tables

**Table 1:** Representative PeLED performance benchmarks reported through September 2026. NR = not reported in the cited source. The near-infrared FAPbI_3_ result is separated from the visible red, green, and blue display benchmarks.

Year/Color/Study	EL Peak/CIE	Peak EQE	Condition at Peak EQE	Practical-Condition Performance	Area/Test Condition	Key Interpretation
2024/pure red/Feng et al. [24]	638 nm/CIE NR	26.1%	NR	16.0% EQE at 1000 cd m^−2^; measured T_50_ 7.5 h at 100 cd m^−2^	Area NR; source-reported steady-state EL	Visible red benchmark; color-stable CsPbI_3_ QD device
2023/green/Bai et al. [25]	EL peak NR/CIE NR	30.84%	6514 cd m^−2^	Average peak EQE 29.05 +/− 0.77%; other operating-condition data NR	Area/drive NR	Visible green efficiency benchmark
2024/blue/Yuan et al. [26]	483 nm/CIE NR	21.4%	22 cd m^−2^	Measured T_50_ 129 min at 100 cd m^−2^	Area/drive NR	Visible blue benchmark; reduced-dimensional emitter
2024/near-infrared/Li et al. [27]	805 nm/CIE N/A	32.0%	20 mA cm^−2^	~30% EQE at 100 mA cm^−2^; measured T_50_ 17 h at 100 mA cm^−2^ and 20 C	Area NR; constant-current stability test	NIR benchmark; not a visible-display red benchmark

**Table 2:** Comparative material matrix for representative 3D, quasi-2D/reduced-dimensional, and nanocrystal PeLED emitters. NR indicates a quantity not reported in the cited source; values are not estimated or back-calculated.

Emitter Class/Representative Study	Composition/Structural Feature	Trap Density/PLQY	Charge-Transport Evidence	Device Performance	Lifetime/Spectral Stability	Primary Advantage/Limitation
3D/Li et al. [27]	FAPbI_3_ + 5AVA/PyNI; increased tetragonal-phase fraction	Trap density NR; PLQY ~96%	Continuous 3D network; EQE remains ~30% at 100 mA cm^−2^	805 nm NIR; peak EQE 32.0%	Measured T_50_ 17 h at 100 mA cm^−2^; spectral drift NR	Direct vertical transport and high radiative efficiency/weaker confinement; NIR-specific benchmark
Quasi-2D/Ma et al. [93]	PEA-based reduced-dimensional perovskite + TFPPO; narrower QW distribution dominated by n ≥ 5	Trap density NR; PLQY NR	Transient-absorption data used to evaluate carrier funneling	~495 nm; peak EQE 25.6%; mean 22.1 +/− 1.2% across 40 devices	Measured half-life 115 min at 7200 cd m^−2^; phase uniformity improved	Strong confinement and energy funneling/phase-distribution and transport sensitivity
Nanocrystal/Kim et al. [129]	~10 nm mixed-cation bromide cores with covalently bound BPA shell	Trap density NR; PLQY NR	Core/shell treatment designed to preserve transport while passivating surfaces	Green; peak EQE 28.9%; max luminance ~470,000 cd m^−2^	Measured T_50_ 520 h at 1000 cd m^−2^; >30,000 h at 100 cd m^−2^ is estimated	Strong confinement and surface control/surface chemistry and ligand-related transport constraints
Reduced-dimensional blue/Yuan et al. [26]	Mixed 2D-3D perovskite with ionic additive	Trap density NR; PLQY NR	Phase and injection control under electrical operation	483 nm; peak EQE 21.4% at 22 cd m^−2^	Measured T_50_ 129 min at 100 cd m^−2^; spectral stability improved vs. control	Efficient blue emission/stability and phase control remain limiting

**Table 3:** Consolidated comparison of the principal device-engineering case studies from Section 3 and Section 4. Control values are listed only when a matched value is reported in the cited study; otherwise, NR or “no matched control reported” is used.

Study/Material	Engineering Intervention/Fabrication/Architecture	Control -> Optimized EQE	Luminance/Voltage	Lifetime Condition	Principal Trade-Off	Evidence Type
Chen et al./multicomponent blue 3D	Pb/Sr all-site alloying; spin-coated PeLED	No matched control EQE reported -> 23.3% at 487 nm (11.9% at 468 nm; 7.5% at 464 nm)	~5700 cd m^−2^ at 487 nm; voltage curve reported	NR	Deeper-blue shift accompanied by lower EQE	Direct CL/PL/device measurements; crystallization mechanism interpreted by source authors
Li et al./3D FAPbI3	Dual-additive crystallization; ITO/ZnO-PEIE/FAPbI_3_/TFB/MoOx/Au	~20% control -> 32.0% optimized	Max radiance 390 W sr^−1^ m^−2^ at 3.6 V	Measured T_50_ 17 h at 100 mA cm^−2^, 20 C	NIR device; mechanism links phase/crystallization to recombination	Direct device/GIWAXS/PL measurements plus source-author mechanistic interpretation
Ma et al./reduced-dimensional	TFPPO-mediated QW-distribution control and passivation	Matched control value NR in manuscript -> 25.6%	Initial luminance 7200 cd m^−2^ for lifetime test; voltage NR	Measured half-life 115 min at 7200 cd m^−2^	Phase control can improve confinement but multiphase transport remains intrinsic	Direct transient-absorption/device measurements plus source-author interpretation
Wang et al./CsPbBr3	CsTFA-mediated nucleation, morphology control, and boundary passivation	1.6% -> 10.5% (mean maximum 1.2% -> 9.4%)	Voltage-dependent EQE reported; luminance NR	Directly measured 15 h -> >250 h at 100 cd m^−2^; epoxy-encapsulated, measured in air	Morphology and chemical passivation change simultaneously	Direct microscopy/device measurements; carrier-redistribution mechanism interpreted by source authors
Chu et al./large-area MAPbI3	Low-temperature blade coating with FPMAI and N_2_-knife drying; ITO/poly-TPD/perovskite/TPBi/LiF/Al	No matched spin-coated device control reported -> 16.1% at 0.04 cm^2^; 12.7% at 1 cm^2^	Luminance/voltage reported in source; key comparison is area scaling	Source reports measured area-dependent operational tests	EQE decreases with larger electrically active area; scalable coating adds process-control demands	Direct film mapping and device measurements
Cao et al./structured FAPbI3	Spontaneously formed submicrometre platelets for internal outcoupling	No matched control EQE reported -> 20.7%	NR	NR	Optical gain from structured morphology can complicate interface deposition and scale-up	Direct microscopy/device data; outcoupling gain partly supported by optical simulation
Kim et al./BPA core–shell nanocrystals	In situ fragmentation and covalent BPA shell formation	No matched control EQE reported -> 28.9%	Max luminance ~470,000 cd m^−2^; voltage NR	Measured T_50_ 520 h at 1000 cd m^−2^; >30,000 h at 100 cd m^−2^ estimated	Added surface chemistry/process complexity vs. improved confinement/passivation	Direct performance/XPS/TEM measurements plus source-author mechanistic interpretation

**Table 4:** Application-readiness comparison for emerging PeLED device concepts. Metrics are reported only when applicable and available in the cited study; NR = not reported; N/A = not applicable.

Application/Representative Study	Resolution/Active Area	Mechanical/Optical Metric	Bandwidth/Data Rate	EQE/Lifetime	Manufacturing/Integration Evidence	Main Readiness Bottleneck
Active-matrix display/Gao et al. [42]	30-ppi green; 90-ppi RGB demonstrations	NR	Response times: tens of microseconds	Representative pixel 23.3%; >20% from 500–3000 cd m^−2^; lifetime NR	Integrated with amorphous-Si TFT backplane	Lifetime, higher pixel density, uniform full-color scale-up
Pixelated QD superlattice/Zhang et al. [238]	Up to 5080 ppi; 1.85-inch active-matrix display	NR	Video playback demonstrated	EQE 30.9%; luminance 117,144 cd m^−2^; lifetime NR	Commercial TFT-backplane integration demonstrated	Yield, operational lifetime, and scalable patterning
Thermally writable device/Bi et al. [239]	Handwritten/stamped patterns; active area NR	EL retained beyond 80 deg bending; repeated deformation reported	NR	Peak EQE ~1.35%; lifetime NR	Localized ~300 C thermal writing on PET/ITO device	High writing temperature; electrically erasable once rather than repeatedly rewritable
Flexible RGB quantum-wire PeLEDs/Cao et al. [250]	1.5 × 1.5 cm^2^ flexible; rigid devices to 3 × 3.5 cm^2^	Bending-cycle metric NR	NR	Blue 12.41%; sky-blue 16.49%; green 26.09%; red 9.97%; lifetime NR	Template-confined all-inorganic quantum wires; top-emission architecture	Long-term bending endurance, lifetime, and manufacturing throughput
Transparent NIR PeLED/Xie et al. [251]	120 mm^2^ functional area	Average visible transmittance > 55%	NR	EQE 5.7% at 5.3 mA cm^−2^; lifetime NR	Transparent Al/ITO/Ag/ITO top electrode	Simultaneous transparency, low resistance, efficiency, and lifetime
Visible-light communication/Tang et al. [253]	Area NR	NR	90 Mbit s^−1^	EQE/lifetime NR	Directly modulated PeLED communication link	Signal stability under ion migration and high-speed/high-current operation
Dual emitter-detector/Bao et al. [254]	7.25 to 0.10 mm^2^ devices	NR	−3 dB EL bandwidth 1.8 -> 21.5 MHz; 1 Mbit s^−1^ intrachip link	Peak EQE 21.2%; lifetime NR	Identical FAPbI_3_ diodes operate as emitter and detector	Operational lifetime, integration density, and reproducibility

## Data Availability

No new data were created or analyzed in this study. Data sharing is not applicable to this article.
